# Membranolytic Mechanism of Amphiphilic Antimicrobial β-Stranded [KL]_n_ Peptides

**DOI:** 10.3390/biomedicines10092071

**Published:** 2022-08-24

**Authors:** Fabian Schweigardt, Erik Strandberg, Parvesh Wadhwani, Johannes Reichert, Jochen Bürck, Haroldo L. P. Cravo, Luisa Burger, Anne S. Ulrich

**Affiliations:** 1 Karlsruhe Institute of Technology (KIT), Institute of Organic Chemistry, Fritz-Haber-Weg 6, 76131 Karlsruhe, Germany; 2 KIT, Institute of Biological Interfaces (IBG-2), P.O. Box 3640, 76021 Karlsruhe, Germany; 3 Laboratório de Biofísica Molecular, Universidade de São Paulo, Ribeirão Preto 14040-901, SP, Brazil

**Keywords:** cationic antimicrobial peptides, length dependent activity, antimicrobial activity, hemolysis, vesicle leakage, solid-state ^31^P-, ^15^N- and ^19^F-NMR, β-stranded peptides, β-sheets, structure and orientation of peptides in membranes

## Abstract

Amphipathic peptides can act as antibiotics due to membrane permeabilization. KL peptides with the repetitive sequence [Lys-Leu]_n_-NH_2_ form amphipathic β-strands in the presence of lipid bilayers. As they are known to kill bacteria in a peculiar length-dependent manner, we suggest here several different functional models, all of which seem plausible, including a carpet mechanism, a β-barrel pore, a toroidal wormhole, and a β-helix. To resolve their genuine mechanism, the activity of KL peptides with lengths from 6–26 amino acids (plus some inverted LK analogues) was systematically tested against bacteria and erythrocytes. Vesicle leakage assays served to correlate bilayer thickness and peptide length and to examine the role of membrane curvature and putative pore diameter. KL peptides with 10–12 amino acids showed the best therapeutic potential, i.e., high antimicrobial activity and low hemolytic side effects. Mechanistically, this particular window of an optimum β-strand length around 4 nm (11 amino acids × 3.7 Å) would match the typical thickness of a lipid bilayer, implying the formation of a transmembrane pore. Solid-state ^15^N- and ^19^F-NMR structure analysis, however, showed that the KL backbone lies flat on the membrane surface under all conditions. We can thus refute any of the pore models and conclude that the KL peptides rather disrupt membranes by a carpet mechanism. The intriguing length-dependent optimum in activity can be fully explained by two counteracting effects, i.e., membrane binding versus amyloid formation. Very short KL peptides are inactive, because they are unable to bind to the lipid bilayer as flexible β-strands, whereas very long peptides are inactive due to vigorous pre-aggregation into β-sheets in solution.

## 1. Introduction

Membrane-permeabilizing amphiphilic peptides are a promising class of antimicrobial agents to combat multidrug-resistant bacteria that are responsible for an increasing number of nosocomial infections [1,2,3]. Antimicrobial peptides (AMPs) are found in almost all types of organisms and constitute a natural host defense system against microorganisms [4,5]. An important goal in biophysical studies of AMPs is to describe their molecular mechanism of action and to understand which factors are important for activity. It is, therefore, necessary to examine not only the biological action of such peptides, such as antimicrobial activity and hemolytic side effects but also their structural properties, especially when interacting with lipid bilayers. Macroscopically aligned membrane samples reveal a wealth of 3D information when studied by oriented circular dichroism (OCD) [6] and solid-state NMR spectroscopy (SSNMR) [7,8,9,10,11].

Many natural and man-made AMPs are cationic and fold into simple amphiphilic α-helices. Much fewer examples exist of another fundamental type of secondary structure, namely amphiphilic β-strands. A classic model sequence, [KIGAKI]_3_-NH_2_, had been designed from alternating polar and hydrophobic side chains [12,13,14]. Recently, we presented an even simpler group of so-called KL peptides, consisting of the alternating (lysine-leucine) dipeptide repeat: [KL]_n_-NH_2_. Recently, four KLx peptides of different lengths (x = number of residues, x = 2n) with 6–18 amino acids were compared in terms of their antimicrobial and hemolytic activities [15]. KL10 had a better antimicrobial effect than KL6, KL14 or KL18. Since KL14 and KL18 were highly hemolytic, KL10 was clearly the most promising antibiotic candidate. These KL peptides showed interesting aggregation characteristics, especially at high pH and/or in the presence of phosphate ions. Aggregation was faster not only at higher concentrations of peptides and phosphate but also for the longer peptides (even when compared at the same mass ratio), and the resulting aggregates were inactive against bacteria [15]. If we want to understand and optimize such peptides further, it is obviously important to take aggregation into account, both in solution and when membrane-bound. For example, we were able to demonstrate that the antimicrobial activity was strongly dependent on the detailed method of performing the minimum inhibitory concentration (MIC) assay because the order of mixing had a pronounced effect on aggregation [15].

In our previous study on four different KL peptides, the length had been varied in steps of four residues, i.e., in increments of about 15 Å (counting 3.7 Å per residue in an extended β-strand), which had given only a very rough estimate of the optimal peptide length. In the present study, we have thus compared 20 peptides to obtain a better length-dependent profile and explore further features (see Table 1). Peptides with lengths from 8 to 16 residues were prepared in increments of single residues, and we furthermore extended the overall range up to 26 amino acids. For odd-numbered peptides, the Lys-to-Leu ratio is no longer 1:1, which changes the balance between charged and hydrophobic residues. To investigate this subtle effect, we further designed some inverted LK peptides, starting with Leu. For example, KL9 ([KL]_4_K) and LK9 (L[KL]_4_) have different charges and hydrophobicity despite their same length.

**Figure 1:**
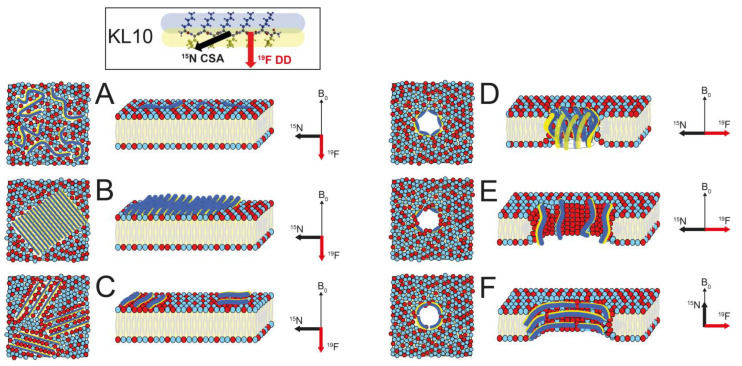
Different possible models of KL peptides in lipid bilayers, showing hydrophobic regions (lipid acyl chains, Leu side chains) in yellow, polar groups (uncharged lipid head groups) in light blue, cationic groups (Lys side chains) in dark blue, and anionic groups (charged lipid head groups) in red. Peptides are depicted in an extended β-strand conformation. The amphiphilic nature of the blue-yellow peptide structure of KL10 is seen in the box at the top. Superimposed on its molecular structure are the orientations of the relevant ^15^N- and ^19^F-NMR tensors as black and red arrows, respectively. The panels on the left show a top view of the membrane, and the central panels illustrate the respective side views. The panels on the right indicate the corresponding orientation of the NMR tensors in ^15^N- and ^19^F-labeled peptides when placed as an oriented membrane sample into the static magnetic field B_0_ (which is aligned along the membrane normal). **Model A:** Monomeric peptides floating around on the membrane surface with high flexibility. **Model B:** Peptides on the membrane surface forming β-sheets that are stabilized by H-bonds between peptides. **Model C:** Peptides on the membrane surface that are indirectly assembled as an immobilized patch via clustering of anionic lipids between the strands. **Model D:** Peptides in a transmembrane orientation forming a β-barrel-type pore that is stabilized by H-bonds between peptides. **Model E:** Peptides forming a toroidal wormhole pore with peptides in a transmembrane orientation, with lipids in between the peptides, such that the pore interior is enriched in anionic lipids. **Model F:** Peptides lining the inside of a β-helix-type pore, with the long axis of the β-strand parallel to the membrane surface.

In addition to the standard functional assays used in our previous study (MIC, hemolysis, vesicle leakage, CD in solution), here we also analyzed the peptide structure and membrane alignment by means of OCD and SSNMR in oriented lipid bilayers. These complementary methods offer detailed information about the peptide conformation, the alignment of its backbone in the membrane, and its molecular mobility. This way, we should be able to discriminate between several conceivable models of membrane-bound structures, as illustrated in Figure 1, in order to find out which of these architectures is responsible for membrane permeabilization. It is reasonable to assume that, upon membrane contact, a monomer gets amphiphilically embedded in the bilayer surface and can diffuse laterally within the plane as a flexible peptide strand (Figure 1A). Such a “2-dimensionally disordered” state has already been demonstrated for the related KIGAKI peptides [14]. Given that the KL and KIGAKI peptides tend to precipitate from aqueous solution as H-bonded amyloid-like fibrils [13,14,15], they might do the same when embedded in a membrane. That is, once the amphiphiles are immersed and pre-ordered in the 2-dimensional plane, they could self-assemble into immobile amphiphilic β-sheets and eventually disrupt the two lipid monolayers due to the difference in lateral pressure in the inner and outer leaflet [16] (Figure 1B). Alternatively, it is conceivable that a number of monomers could induce local lipid clustering and/or trap some lipids in between them, thereby forming a condensed peptide-lipid assembly with largely restricted mobility (Figure 1C). Especially in the presence of anionic lipids, this would lead to lateral phase segregation into patches enriched in anionic lipids and cationic peptides, which could likely lead to membrane permeabilization [17]. In all of these scenarios, the peptide backbone would end up parallel to the bilayer plane, and the hydrophobic side chains would point along the membrane normal. For the NMR discussion below, the geometry of the two orientation-dependent NMR parameters is illustrated in the box at the top of Figure 1. The red vector depicts the alignment of the C_α_-C_β_ bond, which is equivalent to the ^19^F dipole-dipole interaction (DD) of the CF_3_-labeled side chain used here; the black arrow represents the amide ^15^N-^1^H bond vector, which can be taken as the axis of the chemical shift anisotropy tensor (CSA). These two vectors are essentially orthogonal to one another, as is obviously the case for the N-H bonds and side chains in a β-strand and/or β-sheet. Note that the yellow surface of the KL peptide denotes the hydrophobic Leu side chains, while the blue surface stands for the cationic Lys residues. As an example, KL10 is shown in the insert.

If, on the other hand, the KL peptides permeabilize the membrane by assembling into discrete transmembrane pores, the β-strands would have to tilt or flip, and several different architectures are conceivable in that case. H-bonded transmembrane β-barrels, as are characteristic in outer membrane porins, typically consist of alternating polar and hydrophobic residues, just like the KL peptides. Such peptidic assemblies have been suggested for a membrane-bound form of the Alzheimer Aβ-peptide [18,19,20]. In the case of the KL peptides, one would expect the backbone to be strongly tilted or almost upright, depending on the oligomer number (Figure 1D). Alternatively, it is also conceivable that a toroidal wormhole might form, consisting of several monomeric cationic peptides that are separated by anionic lipid head groups (Figure 1E). This pore architecture has been described for the α-helical magainin-family and KIA peptides and was also proposed for the β-hairpin peptide protegrin-1 [21]. In both of these pore models—a β-barrel (model D) as well as a toroidal wormhole (model E)—the KL backbone should be strongly tilted or aligned more or less upright in the membrane, i.e., distinctly different from the surface-bound peptides in models A/B/C.

To complete the range of possibilities, one further conceivable type of backbone alignment could theoretically be constructed from stacks of β-strands (Figure 1F). In this architecture, the orientation of the peptide backbone would once more be completely orthogonal to both A/B/C as well as D/E. In such a hypothetical transmembrane pore, the backbone would be aligned horizontally or slightly tilted, reminiscent of the β-helix of Gramicidin A (though that sequence consists of alternating L- and D-amino acids) [22]. In this model F, the C_α_-C_β_ vector of the side chains would also be aligned in the plane of the membrane, while the amide bond vector would be roughly parallel to the membrane normal. To differentiate between these distinctly different functional models (A/B/C vs. D/E vs. F), the backbone alignment (black amide bond vector) and the side-chain orientations (red arrow) need to be determined. Here, we have performed such analysis using SSNMR in oriented membrane samples by observing ^15^N-labels in the backbone and ^19^F-labels in the side-chains. NMR parameters reflecting the peptide mobility and any length-dependent change in alignment will allow further differentiation between the above-mentioned models A-F. We have thus compiled a battery of KL peptides with different lengths in order to elucidate the mechanism behind their intriguing length-dependent activity. To this aim, we compared their functional activities (antimicrobial, hemolytic, vesicle leakage) and determined their membrane-bound structures (oriented CD, ^15^N-NMR, ^19^F-NMR).

## 2. Materials and Methods

### 2.1. Materials

The lipids 1-palmitoyl-2-oleoyl-*sn*-glycero-3-phosphatidylglycerol (POPG), 1-palmitoyl-2-oleoyl-*sn*-glycero-3-phosphatidylcholine (POPC), 1,2-dimyristoyl-*sn*-glycero-3-phosphatidylglycerol (DMPG), 1,2-dimyristoyl-*sn*-glycero-3-phosphatidylcholine (DMPC), and 1,2-dierucoyl-*sn*-glycero-3-phosphatidylcholine (DErPC) were purchased from NOF Europe (Grobbendonk, Belgium). 1-myristoyl-2-hydroxy-*sn*-glycero-3-phosphatidylcholine (lyso-MPC), 1,2-dimyristoleoyl-*sn*-glycero-3-phosphatidylglycerol (DMoPG), 1,2-dimyristoleoyl-*sn*-glycero-3-phosphatidylcholine (DMoPC), and 1,2-dierucoyl-*sn*-glycero-3-phosphatidylglycerol (DErPG) were obtained from Avanti Polar Lipids (Alabaster, USA). Fmoc-protected amino acids and reagents for peptide synthesis were purchased from Merck Biosciences (Darmstadt, Germany) or Iris Biotech (Marktredwitz, Germany). ^15^N-labelled amino acids for NMR experiments were purchased from Cambridge Isotope Laboratories (Andover, MA, USA) and were Fmoc-protected using Fmoc-Cl as described previously [23]. The ^19^F-NMR label 3-(trifluoromethyl)-l-bicyclopent-[1.1.1]-1-ylglycine (CF_3_-Bpg) was obtained from Enamine (Kiev, Ukraine). Solvents for peptide synthesis were obtained from Merck (Darmstadt, Germany) or Biosolve (Valkenswaard, The Netherlands), and solvents for HPLC purification were obtained from Fischer Scientific (Geel, Belgium). The fluorescent lipid 1,2-dioleoyl-*sn*-glycero-3-phosphoethanolamine-N-(lissamine rhodamine B sulfonyl) (Rhod-PE) was obtained from Avanti Polar Lipids. The fluorescent probes 8-amino-naphtalene-1,3,6-trisulfonic acid sodium salt (ANTS) and p-xylene-bis(pyridinium)bromide (DPX) were purchased from Invitrogen Molecular Probes (Karlsruhe, Germany).

### 2.2. Peptide Synthesis

KL peptides with lengths between 6 and 26 amino acids were synthesized with standard Fmoc solid-phase synthesis methods using a Syro II multiple peptide synthesizer (MultiSynTech, Witten, Germany). Coupling was performed using 4 eq. of the amino acid, 4 eq. HOBt, 4 eq. HBTU, and 8 eq. DIPEA as described previously [15]. ^15^N- and ^19^F-labeled amino acids were coupled manually with a longer coupling time (18 h) using 1.2 to 2 eq. of the labeled amino acids. Peptide purification was done using a JASCO (Groß-Umstadt, Germany) high-pressure liquid chromatography (HPLC) system. Some selected chromatograms of the purified peptides, along with their mass spectra, are shown in Figure S2. A semi-preparative Vydac C18 column was used, with a water/acetonitrile gradient containing 5 mM HCl. An LC-MS system (liquid chromatography instrument from Agilent, Waldbronn, Germany; QTOF ESI mass spectrometer from Bruker, Bremen, Germany) was used to check the identity and purity of the purified peptides, which were found to be at least 95% pure. For ^15^N-NMR, a single ^15^N-labeled Leu residue was incorporated into the position of Leu6. For ^19^F-NMR, a selective CF_3_-reporter group in the form of CF_3_-Bpg was placed into the same position, with minimal perturbation due to its hydrophobic nature [13,24]. For all experiments, the weighed-in amount of peptide was used for preparing stock solutions and calculating the concentrations. Note that in every series of samples within any one assay, we used the same weight of peptide, i.e., a constant mass-to-mass peptide-to-lipid ratio, as described previously [15]. Only for visualization and ease of interpretation have we stated in several cases a molar peptide-to-lipid ratio, which we defined for KL14.

### 2.3. Circular Dichroism Spectroscopy (CD)

#### 2.3.1. Sample Preparation

For CD measurements in liposomes, stock solutions of the lipids (5 mg/mL in CHCl_3/_MeOH 1/1 vol/vol) and of the lyophilized peptides (1 mg/mL in H_2_O/MeOH 1/9 vol/vol) were prepared. The final KL samples for CD measurements in pure water were obtained by diluting aliquots of the peptide stock solutions with deionized water, resulting in a peptide concentration of 0.1 mg/mL (27–113 µM, depending on the molecular weight of the peptide). POPC/POPG (1/1 mol/mol) and DMPC were chosen as lipid systems for the CD measurements in liposomes. The lipid concentration in the sample was adjusted to 1 mg/mL to get an analyzable CD signal of the peptides. Merging the peptide and lipid molecules on a molecular level without preceding aggregation of peptides was achieved by mixing suitable aliquots of the organic peptide and lipid stock solutions (co-solubilization). For KL14, a molar peptide-to-lipid ratio (P/L) of 1/20 was chosen, corresponding to a concentration of 65 µM in DMPC (or 75 µM in POPC/POPG 1:1) for the peptide and 1.3 mM or 1.5 mM for the lipid, respectively. The same corresponding peptide-to-lipid mass-to-mass ratio was used for all other KL peptides in order to keep the charge ratio constant, resulting in differing molar P/L ratios. After mixing the organic peptide/lipid solutions and subsequent evaporation of the organic solvents under N_2_ gas flow, the residual peptide/lipid film was vacuum-dried for 4 h and re-dissolved in H_2_O. Then, the samples were vortexed for 5 min and freeze/thaw-cycled tenfold. In a final step, the solutions were treated in an ultrasonic bath (UTR 200, Hielscher, Germany) for 16 min to generate small unilamellar vesicles (SUVs), which were stored overnight at room temperature.

Before the measurement of one series of aqueous peptide solutions, the samples were kept at the slightly acidic pH (~5–6) produced by the residual HCl from HPLC purification in the lyophilized peptides. In a second series of aqueous peptide solutions, and also for the liposome dispersions, the pH was set to 9–10 by adding a small aliquot (2–3 µL) of 0.1 M NaOH to each sample. The pH of each sample was checked with indicator paper.

#### 2.3.2. Measurements

The CD measurements were conducted using a J-815 circular dichroism spectropolarimeter (Jasco, Groß-Umstadt, Germany). Spectra were recorded in the spectral range from 260–190 nm, at 0.1 or 0.5 nm intervals with a scanning speed of 10 nm/min and a spectral bandwidth of 1 nm as described earlier [14]. Samples were measured in a 1 mm quartz glass cuvette (110-QS, Hellma Analytics, Müllheim, Germany) at 30 °C. All KL peptides were measured in pure water and in the presence of small unilamellar vesicles (SUVs) composed of DMPC or POPC/POPG (1/1 mol/mol). Additional details are given in the Supplementary Materials.

### 2.4. Oriented CD

To examine the membrane alignment and conformation of the KL peptides in different lipid systems, oriented CD (OCD) experiments were performed on a Jasco J-810 spectropolarimeter with an OCD cell built in-house [6]. The samples were prepared from peptide stock solutions in MeOH/H_2_O (9/1 vol/vol) with a concentration of 1 mg/mL and lipid stock solutions in MeOH/CHCl_3_ (1/1 vol/vol) with a concentration of 5 mg/mL. The chosen lipid systems were POPC/POPG (1/1 mol/mol) and DMPC/lyso-MPC (2/1 mol/mol). The spectra were taken at wavelengths from 260–185 nm at 8 rotation angles (0°, 45°, 90°, …, 315°) with a scanning speed of 10 nm/min and a spectral bandwidth of 1 nm. Two spectral scans were performed at each rotation angle. These measurements at different rotation angles were done to avoid artefacts caused by linear dichroism. The final spectrum was obtained by averaging all spectra of all angles and baseline zeroing them at 260 nm. More details are given in the Supplementary Materials.

### 2.5. MIC (Minimum Inhibitory Concentration) Assay

The antimicrobial activity of the KL peptides was determined with a MIC assay. Peptide activity was tested against Gram-negative *Escherichia coli* (DSM 1116) and *Enterobacter helveticus* (DSM 18390) and against Gram-positive *Bacillus subtilis* (DSM 347) and *Staphylococcus xylosus* (DSM 20287) as previously reported [15]. Our standard MIC assay [14,25] was modified to avoid unnecessary exposure of peptides to the phosphate-containing growth medium, which was previously found to affect the results due to phosphate-induced aggregation of peptides [15]. Details can be found in the Supplementary Materials.

### 2.6. Hemolysis Assay

Hemolytic activity was examined with a serial 2-fold dilution assay as described earlier [15,26]. Details of the assay are described in the Supplementary Materials.

### 2.7. Vesicle Leakage Assay

For the leakage experiments [27,28], multilamellar vesicles (MLVs) were prepared in buffer containing the fluorophore ANTS, the quencher DPX, 50 mM NaCl, and 10 mM HEPES (pH 7.5). MLVs were extruded 41 times through a Nuclepore polycarbonate membrane with a pore size of 100 nm (Whatman GE Healthcare Europe, Freiburg, Germany) to obtain large unilamellar vesicles (LUV). Dye outside LUVs was removed by gel filtration through spin columns filled with Sephacryl 100-HR (Sigma-Aldrich, Taufkirchen, Germany). Before use, columns were equilibrated with elution buffer (150 mM NaCl, 10 mM HEPES, pH 7.5).

Leakage of entrapped ANTS was monitored by fluorescence dequenching of ANTS [29]. Fluorescence measurements were performed at 30 °C on a FluoroMax2 spectrofluorometer (HORIBA Jobin Yvon, Unterhaching, Germany) with an excitation wavelength of 355 nm and an emission wavelength of 515 nm. The sample was placed in a 10 mm × 10 mm quartz glass cuvette, and constant stirring was applied during the measurement. LUV and peptide solutions were diluted with elution buffer to obtain 100 µM lipids and the wanted peptide-to-lipid ratio in the sample. The level of 0% leakage corresponded to the fluorescence value immediately after the addition of vesicles, while 100% leakage was the fluorescence value obtained after the addition of Triton-X100, 10 min after the addition of vesicles. Additional details about the assay are given in the Supplementary Materials.

### 2.8. Leakage of FITC-Dextrans

The size of lesions in the membrane induced by the peptides was investigated using a modified leakage assay based on the fluorescence quenching of FITC-labeled dextran polymers of different sizes [30,31]. In short, POPC/POPG (1:1) vesicles were prepared with carboxyfluorescein and different size fluorescein isothiocyanate (FITC)-dextrans (FDs, Sigma-Aldrich, Taufkirchen, Germany) inside. When the FDs leak out, the fluorescence signal is quenched by anti-FITC antibodies (SouthernBiotech, Birmingham, AL, USA). Leakage was followed for 30 min, then Triton-X100 was added to completely destroy the vesicles and obtain 100% leakage. The size of the FITC-dextrans was determined by dynamic light scattering (Zetasizer Nano S, Malvern Instruments Ltd., Malvern, UK), as described in the Supplementary Materials.

### 2.9. Solid-State NMR

Macroscopically oriented NMR samples were prepared by co-dissolving appropriate amounts of peptides and lipids and spreading the solution onto thin glass plates. The peptide-to-lipid ratio (P/L) is given in mol/mol. For all samples and sample types, the P/L ratios were calculated for KL14, and the same peptide-to-lipid mass ratios were used for the other peptides to keep the charge ratio constant. All ^15^N-, ^19^F-, and ^31^P-NMR solid-state NMR measurements were carried out on a Bruker Avance 500 or 600 MHz spectrometer (Bruker Biospin, Karlsruhe, Germany) at 308 K, as previously reported [32,33,34,35,36,37]. More experimental details are given in the Supplementary Materials.

#### NMR Data Analysis

^31^P-NMR spectra give information about the phospholipids. The line shape is sensitive to the lipid phase [38], and the degree of orientation of macroscopically oriented samples can also be determined [39]. In our oriented samples, the membrane normal is usually oriented parallel to the external magnetic field B_0_ (the sample tilt is 0°). In a well-oriented sample, there should then be one oriented peak around 30 ppm, but in less oriented samples, there is also a broad powder line shape with a maximum of around −15 ppm. The degree of orientation can be determined by integration.

In oriented samples, the ^15^N-NMR chemical shift of peptides labeled with ^15^N at the backbone amide gives information about the approximate orientation of the ^15^N-^1^H bond. A peak around 200 ppm indicates that the ^15^N-^1^H bond is oriented parallel to B_0_, and a peak around 90 ppm indicates that the ^15^N-^1^H bond is oriented perpendicular to B_0_. In a sample with a 0° tilt, a peak at 90 ppm indicates that the bond is in the membrane plane, and a peak at 200 ppm indicates that the bond is along the membrane normal [40,41].

In the ^19^F-NMR spectrum of CF_3_-Bpg labeled peptides, the dipolar couplings lead to a triplet signal, and the coupling strength gives information about the orientation of the C-CF_3_ bond with respect to the external magnetic field B_0_ [34,42]. In our oriented samples, the membrane normal is usually oriented parallel to B_0_ (the sample tilt is 0°). The sign of the dipolar couplings can also be determined from the spectrum [42]. If the coupling is close to the maximum value of +16 kHz, then the C-CF_3_ bond is parallel to B_0_. If the coupling is close to the minimum value of −8 kHz, then the C-CF_3_ bond is perpendicular to B_0_ [42,43,44]. A coupling close to −8 kHz can also be found in samples where the peptides aggregate and a powder pattern is obtained in ^19^F-NMR; in this case, the coupling will be the same if the oriented sample is rotated so that the membrane normal is perpendicular to B_0_ (the sample tilt is 90°). If, on the other hand, the coupling is scaled by a factor −0.5 upon changing the sample tilt from 0° to 90°; this is a sign of fast rotational diffusion of peptides in the membrane [34].

## 3. Results

### 3.1. Peptide Synthesis

Twenty different peptides consisting of a repetitive Lys-Leu motif were used in this study, with lengths between 6 and 26 amino acids. Peptides starting with Lys are called KLx peptides, and those starting with Leu are called LKx peptides, where x is the total number of amino acids in the sequence. Further analogues of KL10 and KL14 were synthesized for NMR analyses, either with a non-perturbing ^15^N-NMR label at the backbone amide of Leu-6 or with Leu-6 replaced by CF_3_-Bpg for ^19^F-NMR experiments. All peptides were amidated at the C-terminus, and all sequences are listed in Table 1.

### 3.2. Circular Dichroism (CD)

**Figure 2:**
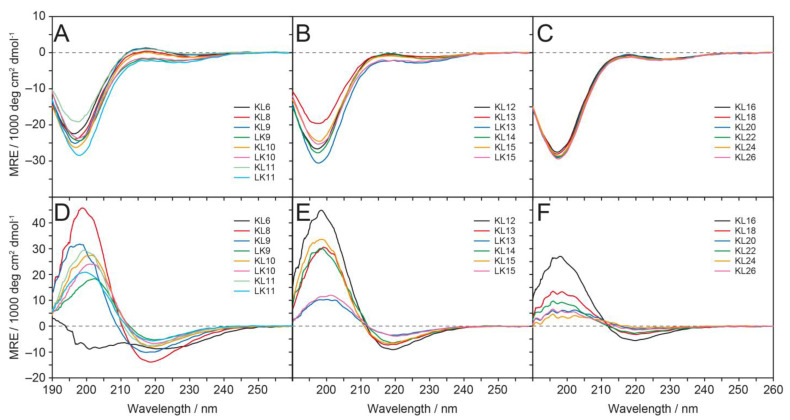
Circular dichroism spectra of KL and LK peptides (including KL6, KL10, KL14 and K18 from ref. [15]). (**A**–**C**) CD spectra in pure water at pH ≈ 5–6, with a peptide concentration of 0.1 mg/mL. (**D**–**F**) CD spectra in the presence of POPC/POPG (1/1) SUVs at pH ≈ 9–10 (HPLC-purified samples were made more basic by adding a small aliquot of 0.1 M NaOH). The lipid concentration was 1 mg/mL (1.3 mM), and the peptide concentration for KL14 was 0.13 mg/mL (65 µM), resulting in a peptide-to-lipid (P/L) molar ratio of 1/20, which provides enough bilayer surface for binding. The other samples were prepared with the same peptide-to-lipid mass-to-mass ratio in order to keep the total weight of peptide material as well as the charge ratio (Lys residues per anionic lipid) constant. The charge ratio of 7 cationic Lys residues per 10 anionic POPG lipids ensures that there is sufficient electrostatic attraction for all peptides to bind to the vesicles.

CD spectroscopy was used to investigate the secondary structure of all KL and LK peptides in solution. In slightly acidic water at pH ≈ 5–6 (Figure 2A–C), the CD line shapes indicated that all peptides were unstructured (random coil spectra), with a minimum close to 197 nm. Phosphate buffer (PB) pH 7.0, which is well established in CD spectroscopy for measurements in aqueous environments, has to be avoided by all means because it will induce strong aggregation of KL into β-sheets that precipitate [15]. In the presence of anionic POPC/POPG (1/1) lipid vesicles in aqueous suspension at pH ≈ 9–10 and P/L = 1/20, (Figure 2D–F), almost all peptides showed a transition to a β-pleated conformation, as indicated in the spectra by a maximum at approximately 198 nm and a minimum close to 219 nm. The only exception was KL6, which was mostly disordered with a non-canonical line shape in the presence of vesicles. The intensity of the CD spectra was significantly reduced for the very long peptides (KL18 and longer), which had a strong tendency to precipitate as β-pleated aggregates. The reason is that aggregation resulted in turbid solutions, in which macroscopic particles would sediment and no longer contribute to the signal. Moreover, even for the shorter KL peptides, one cannot exclude significant differential light scattering and absorption flattening artifacts [45], which are also known to occur at increased turbidity. Therefore, no quantitative secondary structure estimation was attempted.

Further CD analyses were performed on KL and LK peptides in more alkaline water at pH ≈ 9–10 and in the presence of uncharged DMPC vesicles (cf. Figure S3, Supplementary Materials). In aqueous solution at this pH, where the N-terminal amino group is deprotonated and contributes to the polarity and total charge, the shortest peptides, KL6 and KL8, remain mostly unstructured. KL10 exhibits a mixture of random coil and β-pleated signals, while all other peptides show predominantly β-sheet structures. These spectra resemble those in small unilamellar DMPC vesicles dispersed in water at pH ≈ 9–10, where maxima at 195 and minima at 215 nm are found; i.e., there seems to be only a moderate induction of additional β-pleated structure due to the zwitterionic DMPC lipid. We note that in the negatively charged POPC/POPG vesicles discussed above, the β-sheet maxima and minima are shifted towards 198 and 219 nm, respectively, which seems to be an indication of higher aggregation tendency. In contrast, the binding and lipid-induced β-stranded conformation seems to be less pronounced in uncharged zwitterionic membranes, and the signal height is similar or somewhat increased compared to the spectra in water with the pH adjusted to 9–10 in the absence of lipids.

### 3.3. Oriented CD

Oriented CD (OCD) measurements of the KL peptides were performed in macroscopically aligned POPC/POPG (1/1) and DMPC/lyso-MPC (2/1) lipid systems. The idea behind these experiments was to elucidate whether changes in the OCD line shape would reveal any preferred orientation of the β-strands either parallel to the oriented lipid bilayer plane (see Figure 1A–C,F), strongly tilted (Figure 1D), or essentially parallel to the membrane normal (Figure 1E). Usually, OCD is applied only to α-helical peptides, where it gives straightforward information on the approximate helix tilt angle in oriented membrane samples [6,46,47]. Only very few attempts have been made so far to characterize the directional dependence of the CD bands of β-stranded and/or β-sheet structures in lipid bilayers. Note, moreover, that it is not straightforward to discriminate a local β-stranded conformation from an oligomeric β-sheet. Only in the case of poly[Leu-Lys] bound to phosphatidylcholine was it shown that the long-wavelength negative band is stronger in OCD than that in the isotropic CD spectrum, while the short-wavelength positive band is weaker, which was interpreted in terms of a backbone orientation parallel to the surface of the bilayers [48]. However, the β-sheet CD tensor is far more complex than that of an α-helix [49], and as there are numerous structural variations of β-pleated conformations (e.g., parallel β-sheets, antiparallel β-sheets, twisted amyloid fibrils, β-helices, etc.), no comprehensive theory or evaluation procedure exists. Nonetheless, here we tried to apply OCD to the KL peptides in the above-mentioned oriented bilayers in order to determine whether the spectral pattern of OCD spectra is different from that of the isotropic spectrum, which would at least indicate some orientational preference in the membrane, allowing to exclude some of the models in Figure 1.

**Figure 3:**
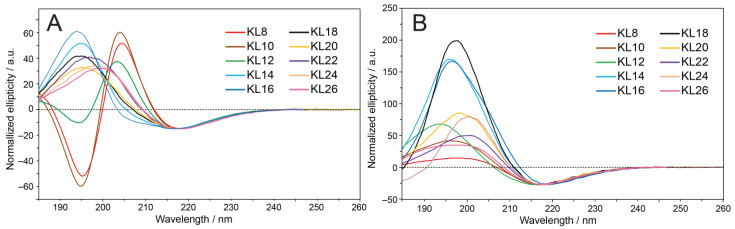
Oriented CD (OCD) spectra of KL peptides with an even number of residues. The P/L of KL14 was 1/20, and the same mass ratio was used for the other peptides. Spectra are normalized to the intensity of KL14 at 217 nm. (**A**) In POPC/POPG (1/1), all peptides show a minimum close to 217 nm, indicating extended β-strands or complete β-sheet formation. The shortest peptides KL8, KL10 and KL12 also have another minimum at approximately 195 nm, suggesting a partially unstructured conformation. (**B**) In DMPC/lyso-MPC (2/1), all peptides show a minimum at 217 nm and very broad positive bands, indicating β-sheet formation, given, for the longer peptides, strong absorption flattening artifacts due to aggregation.

For better comparison, all OCD spectra in Figure 3A,B have been scaled to the negative band of KL14 at approximately 217 nm. The lipid bilayers were oriented on a solid quartz glass support and kept hydrated at 97% relative humidity; i.e., the membranes are essentially fully hydrated, but there is no excess water present. Peptides with an even number of amino acids from 8 to 26 were examined. In the POPC/POPG lipid system, an OCD minimum was found at approximately 217 nm. A maximum is seen between 194 and 199 nm for peptides longer than KL12, indicating that they form β-pleated structures in these membranes under OCD conditions. The longer peptides have characteristic β-strand/-sheet spectra with quite broad positive bands. These exhibit distinct absorption flattening artifacts [45], indicating a high tendency to form aggregates, in which the β-strands seem to have no preferred alignment.

Generally, for the KL peptides, a comparison of oriented OCD and conventional isotropic CD spectra is difficult due to the high aggregation tendency at P/L = 1/20 (lower P/L ratios could not be measured due to low sensitivity). If one considers the ratio of the positive and negative bands for KL14 and KL16, where absorption flattening artifacts are less pronounced, only small differences compared with the liposome spectra can be found. This means that no preferred alignment of the β-strands can be identified in these oriented OCD samples. Interestingly, in POPC/POPG bilayers, the shortest peptides with 8–12 amino acids exhibit an additional minimum at approximately 195 ppm, revealing that these peptides have a significant fraction of an unordered conformation under OCD conditions. Either a sub-population of these peptides is bound to the membrane as fully assembled β-sheets, while the residual fraction is not bound and very mobile with no defined structure, or all peptides are bound to the membrane but not fully extended in a straight β-stranded conformation.

Finally, a highly informative DMPC/lyso-MPC (2/1) lipid system was included, which exhibits a highly positive spontaneous curvature. The intrinsic monolayer curvature of this lyso-lipid mixture is known to drive α-helical KIA peptides into stable transmembrane pores of the toroidal wormhole type [50,51]. For the KL peptides, however, we find that the OCD spectra in DMPC/lyso-MPC are very similar to those in POPC/POPG, with a minimum at 217 nm. Despite the distinct characteristics in charge and curvature, the only difference compared to POPC/POPG can be seen for very short KL peptides, which give characteristic β-sheet signals with no minimum at 195 nm. However, the positive band around 200 nm is rather small, which can be due to a random coil contribution, which is largest for KL8 and reduced for KL10 and KL12. KL14, KL16 and KL18 have higher positive bands than in POPC/POPG, while the even longer peptides again demonstrate clear signs of aggregation, with very broad positive bands and pronounced absorption flattening artifacts reducing the band around 200 nm.

### 3.4. Antimicrobial Activity

The activity of KL and LK peptides against bacteria was examined using a MIC assay. In these two-fold dilution series, any values differing by a factor of two are not considered to be significantly different. We have previously shown that KL peptides, especially the longer ones, aggregate vigorously in the presence of phosphate ions [15]. We also demonstrated that the standard MIC assay that is routinely used in many laboratories is biased because peptides are exposed to the phosphate-containing medium before coming in contact with the bacteria. Therefore, here we used a modified MIC assay that was developed in our previous study [15], as described in the Methods section. In this approach, a two-fold dilution series was first prepared with peptides dissolved in water, and only thereafter were the bacteria in the medium added so that the peptides were simultaneously exposed to bacteria and phosphate-containing medium.

As seen in Table 2, there is a clear correlation between peptide length and biological activity, with the highest activity for peptide lengths of approximately 12 amino acids. KL6 was always inactive, in line with the observation above by CD that it does not form any β-strands in the presence of membranes. Regarding the even-numbered KL peptides, KL8 is somewhat antimicrobially active, KL10 and KL12 show the highest activity, while KL14 is moderately active, and KL16 and longer peptides are significantly less active. The odd-numbered KL peptides are slightly more active than the KL peptides with one less amino acid, but the values lie within a factor of two. The inverted sequence LK9 shows activity similar to that of KL9, LK10 is similar to KL10, and LK11 is similar to KL11. For the particular lengths 13 and 15, the charge-dominated KL peptides are more active (with 4–8 times lower MIC values) than the hydrophobicity-dominated LK peptides.

### 3.5. Hemolysis

Amphipathic antimicrobial peptides not only show membranolytic effects against bacteria but can also permeabilize eukaryotic cells such as erythrocytes. The hemolytic activities of the KL peptides were recorded for several different peptide concentrations and are summarized in Table 2 as HC_50_ values (concentration of peptide giving 50% hemolysis). The complete graphs of hemolysis as a function of peptide concentration are shown in Figure S4. We see that the KL peptides can be highly hemolytic, and the general trend is that hemolysis increases with length. Only KL6, KL8, KL9, and KL11 have very low hemolytic activity. KL10 gave 20% hemolysis at 8 µg/mL, KL11 gave almost no hemolysis, and KL12 gave 50% or more hemolysis at 8 µg/mL. Peptides with 14 or more amino acids gave 50% or more hemolysis even at 2 µg/mL. The trend seen above with regard to MIC values, i.e., that longer peptides become less active again, is clearly not observed for the HC_50_ values of hemolysis. Only the concentration-dependent curves show an apparent reduction of hemolysis at higher concentrations for the longer peptides (Figure S4) due to rapid aggregation in solution before the peptides could damage the erythrocytes.

LK peptides with an odd number of residues are found to be more hemolytic than KL peptides of the same length. This difference can be simply attributed to their increased overall hydrophobicity, which is generally known to promote hemolysis [52,53] (as odd-length LK peptides have one excess Leu residue, whereas odd-length KL peptides carry one extra Lys). Interestingly, in the Leu/Lys-balanced pair of 10 amino acids in length, the hemolytic activity turns out to be the other way around: KL10 is considerably more hemolytic than LK10. This finding seems to reflect the more pronounced amphiphilic character of an extended KL10 molecule, which carries a doubly charged Lys at the N-terminus and an entirely hydrophobic C-terminus with an amidated Leu.

### 3.6. Vesicle Leakage

The intriguing length-dependent biological activities raised the question of whether the window of optimal antibiotic activity for peptides KL10 to KL13 might be attributed to the concept of hydrophobic mismatch. Namely, when these peptides are fully extended as β-strands, they have a length of 37 to 48 Å, which seems perfectly matched to span the hydrophobic thickness of a membrane as a transmembrane pore, as illustrated for the two models in Figure 1D,E. It can be generally assumed that the activity of pore-forming peptides will show a distinct dependence not only on peptide length but also on membrane thickness. That is, in a toroidal wormhole and possibly even in a β-barrel, the amphiphilic unit has to be long enough to span the hydrophobic region of the bilayer; otherwise, it should remain inactive. Second, this threshold length should vary when its activity is being compared in membranes of different thicknesses. We thus set forth to determine whether hydrophobic matching between the length of the KL backbone and the bilayer thickness plays a role, as had been previously demonstrated for the amphiphilic α-helical KIA peptides [54].

For these experiments, it was imperative to vary the membrane thickness, which had not been possible in the MIC and hemolysis assays above, because the acyl chain composition of living cells obviously cannot be controlled. Therefore, we performed complementary in vitro experiments by measuring the leakage of a fluorescent dye from small unilamellar vesicles with different well-defined bilayer thicknesses (and comparable spontaneous lipid curvature). Different synthetic lipids were chosen with distinctly different acyl chain lengths (all of them being unsaturated so that they could be measured and compared in the liquid crystalline phase). In all cases, a 1/1 (mol/mol) mixture of zwitterionic phosphatidylcholine (PC) and anionic phosphatidylglycerol (PG) head groups was used to attract the cationic peptides electrostatically to the vesicles and promote complete binding. Furthermore, anionic lipids are known to be one of the main components of bacterial membranes, which in some cases contain more than 50 mol% PG, and also contain other anionic lipids like cardiolipin [55]. Vesicles were prepared in 10 mM HEPES buffer at pH = 7.5.

**Figure 4:**
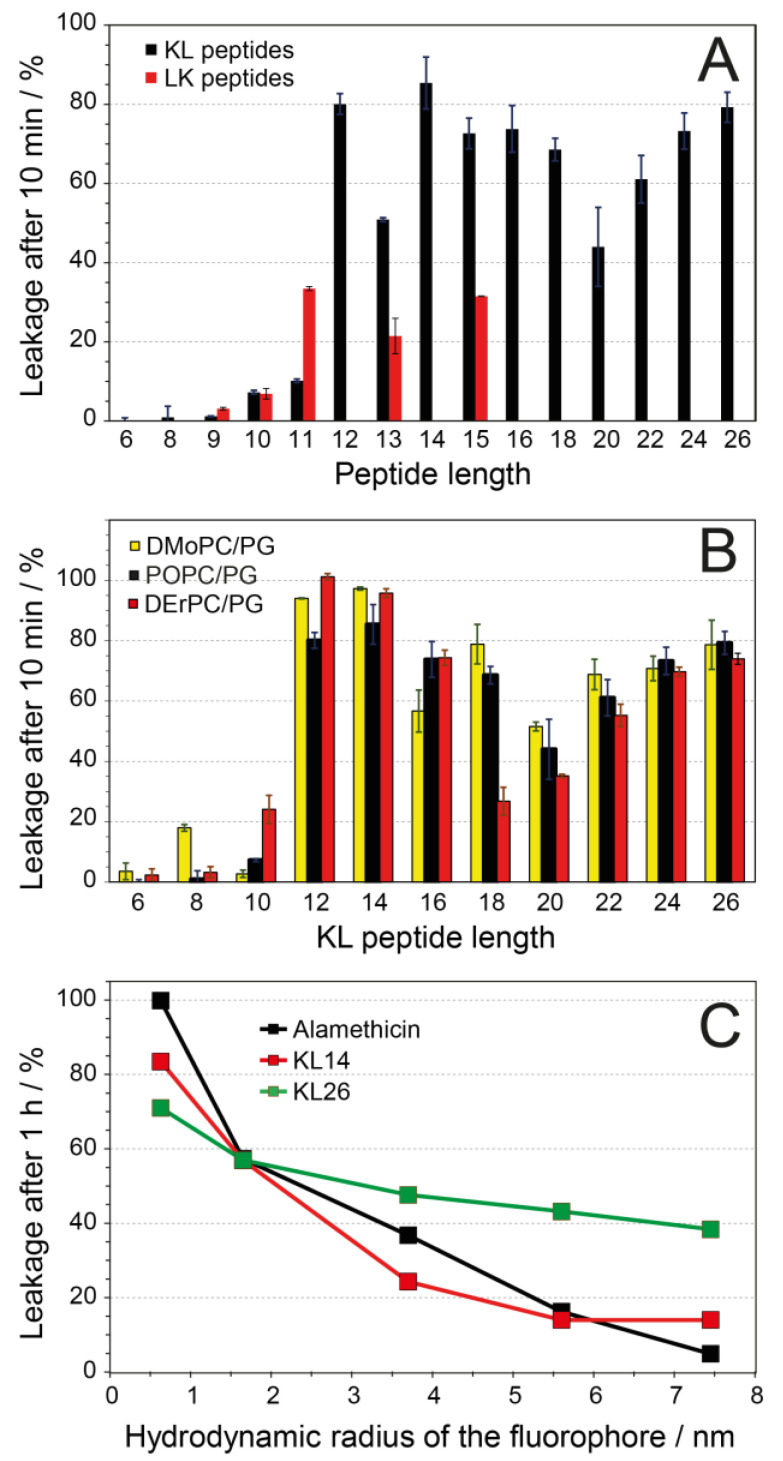
Fluorescent leakage assays of KL peptides in small unilamellar lipid vesicles. (**A**,**B**) For ANTS/DPX leakage, a P/L ratio of 1/20 was set for KL14, and the same peptide-to-lipid mass ratio was used for all other peptides. Leakage was monitored for 10 min after mixing peptides and vesicles. The 100% leakage signal was determined by the addition of Triton X-100. (**A**) Comparison of KL (black) and LK (red) peptides of different lengths in POPC/POPG (1/1) vesicles. (**B**) Leakage of even-numbered KL peptides in lipid systems with different bilayer thickness. Even though we see a length dependence, with a minimum length of 12 amino acids required for leakage to occur, a comparison of all three lipid systems shows that there is essentially no dependence on membrane thickness. (**C**) Leakage of FITC-dextrans of different sizes by KL14 and KL26 in POPC/POPG vesicles. Alamethicin (20 amino acids length) with a published pore size of 1.8 nm was used as a reference peptide. The lipid concentration was 100 µM, and the concentrations of KL14, KL26 and alamethicin were 12, 8 and 2.5 µM, respectively. These concentrations were chosen to obtain a similar leakage for FD4 for each peptide. For KL14 and KL26, the same peptide-to-lipid mass ratio was used. The hydrodynamic radii of the FITC-dextrans were determined by dynamic light scattering assuming spherical particles (see Supplementary Materials for more details).

In POPC/POPG (1/1) vesicles at P/L = 1/20 (Figure 4A), we see hardly any leakage for KL6 to KL11, but KL12 gives almost full leakage within 10 min. KL14 is similar to KL12, and the longer peptides are slightly less active but generally give >50% leakage. Unlike the sharp threshold seen here for the KL peptides (i.e., between KL11 and KL12 in POPC/POPC with C16/C18 acyl chains), the LK peptides do not show such a pronounced length-dependent jump in activity. They also give a lower overall leakage, with a maximum of about 35% for peptides with 11 or more amino acids. Interestingly, LK11 causes essentially the same leakage as LK15, whereas KL11 gives much less leakage than KL15 and less than LK11.

In contradiction to our hydrophobic mismatch hypothesis, it turned out that in thin DMoPC/DMoPG vesicles with short acyl chains (14 carbons, 19.2 Å hydrophobic thickness [56]) and in thick DErPC/DErPG vesicles with very long acyl chains (22 carbons, 34.4 Å hydrophobic thickness [57]), almost the same extent of leakage as in POPC/POPG was found for KL peptides with an even number of amino acids (Figure 4B). In all cases, peptides with up to 10 amino acids give only very little leakage, while KL12 gives almost full leakage, and longer peptides also give a high degree of leakage. Therefore, we can conclude that leakage as a function of peptide length is essentially independent of membrane thickness. It only varies with the peptide length as such but shows no relation to the actual bilayer thickness. This result is in stark contrast to our earlier observations on α-helical KIA peptides with different lengths, where the relative length of peptides compared to the membrane thickness was critically important for function. Those KIA peptides are only active when they are long enough to span the membrane, and this threshold length varies linearly with the thickness of the lipid bilayer [54]. It, therefore, seems that the β-stranded KL-type peptides use a membrane permeabilization mechanism that is completely different from that of α-helical KIA peptides. The KIA peptides have been demonstrated to form toroidal wormhole pores with peptides in a membrane-spanning orientation [50,51], but for the KL peptides, neither this model (Figure 1E) nor the H-bonded β-barrel (Figure 1D) is supported by our leakage experiments.

Next, a modified leakage assay based on the fluorescence quenching of fluorescein-labeled dextran polymers with different sizes [30,31] was used to assess the diameter of the lesions in the membrane that are responsible for leakage. Here, we use the term “lesion” to describe any defects in the membrane that allow molecules to pass through without inferring any mechanism or structure. Carboxyfluorescein and FITC-dextrans with different diameters were trapped inside vesicles, and when leakage was induced by one of the KL peptides, the escaping fluorophores were quenched by specific antibodies outside the vesicles. KL14 and KL26 were used in this experiment to represent different peptide lengths, as they both showed high leakage in the ANTS/DPX assay. As a control, the helical antimicrobial peptide alamethicin was used, which is known from the literature to form water-filled barrel-stave pores with an inner diameter of 1.8 nm in DLPC and 2.6 nm in DPhPC [58,59]; similar-size pores might be expected in our POPC/POPG lipid system. As seen in Figure 4C, the leakage induced by KL14 or KL26 is reduced for the larger dextran sizes. This observation suggests that the KL peptides form lesions of limited size and do not dissolve the membrane completely. The size-dependent curve for KL14 was rather similar to that of alamethicin, indicating that lesions of similar size are formed by the two peptides. This result, however, does not imply that KL14 forms barrel-stave pores like the helical alamethicin. KL26 gave much more leakage for the larger dextrans than did KL14 or alamethicin, so we conclude that it forms larger lesions and less specific damage in the membranes than alamethicin.

### 3.7. Solid-State NMR

To determine the backbone orientation of the membrane-embedded KL peptides, solid-state ^15^N- and ^19^F-NMR experiments were performed on the KL10 peptide in macroscopically oriented samples, carrying either a selective ^15^N-label (^15^N-Leu) or a single ^19^F-labeled amino acid (CF_3_-Bpg) at the position of Leu6. These oriented samples are similar to the OCD samples in terms of composition, preparation and hydration, so we can infer from the corresponding OCD observations that the peptides must also form β-strands in the NMR samples. From previous studies of α-helical peptides, it is known that POPC/POPG (1/1), due to its negative spontaneous curvature, prefers to accommodate molecules within the headgroup region. Amphiphilic peptides thus tend to stay surface-bound in these unsaturated bilayers, maintaining an alignment parallel to the membrane surface. Bilayers with positive, spontaneous curvature, on the other hand, favor the insertion of peptides into the membrane. Therefore, we also used DMPC/lyso-MPC (2/1) for comparison. As a third lipid system with intermediate spontaneous curvature close to zero, we also included DMPC.

**Figure 5:**
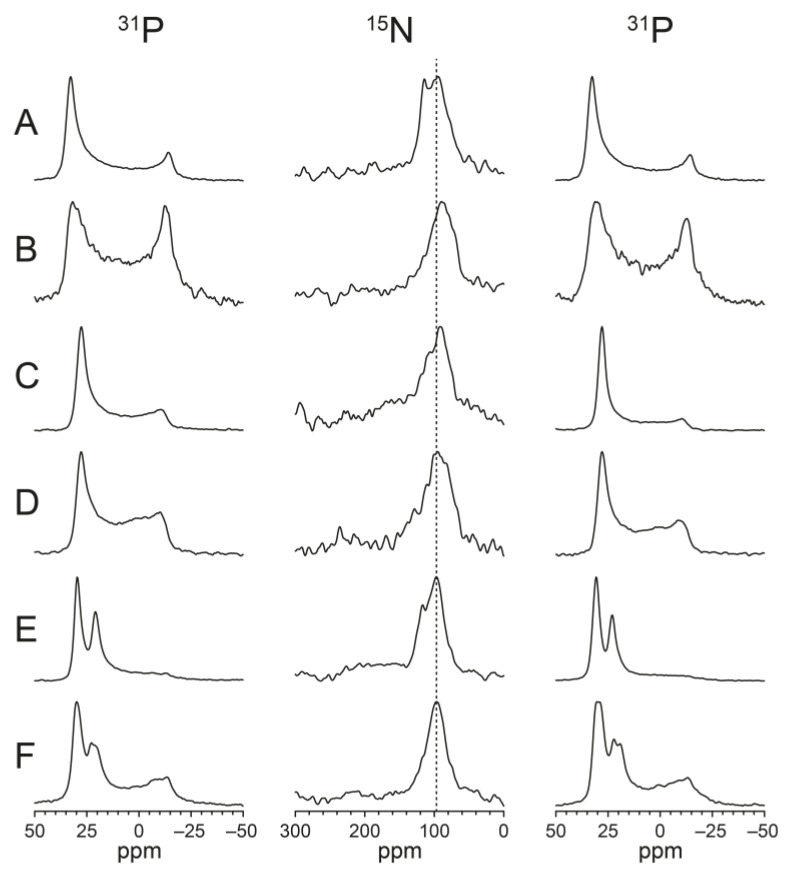
Solid-state NMR data on ^15^N-labeled KL10 embedded in macroscopically aligned membranes with the sample normal aligned parallel to the static magnetic field direction. The middle column shows the ^15^N-NMR spectra. ^31^P-NMR spectra before (on the left side) and after ^15^N-NMR experiments (on the right side) are also given. The lipid systems are (**A**) DMPC, P/L = 1/50; (**B**) DMPC, P/L = 1/20; (**C**) POPC/POPG (1/1), P/L = 1/100; (**D**) POPC/POPG (1/1), P/L = 1/50; (**E**) DMPC/lyso-MPC (2/1), P/L = 1/50; (**F**) DMPC/lyso-MPC (2/1), P/L = 1/20. (P/L ratios are for KL14, and the same peptide-to-lipid mass ratios were used for KL10).

Oriented ^15^N-NMR spectra are shown in Figure 5. Measurements were performed at different peptide concentrations, with P/L ratios from 1/100 to 1/20. ^31^P-NMR spectra were measured before and after the ^15^N-NMR experiments in order to monitor the orientational quality and stability of the samples. A ^31^P-NMR signal at approximately 30 ppm corresponds to the well-oriented portion of the lipid bilayers, while a broad component with a maximum around −15 ppm arises from unoriented phospholipids. In DMPC (Figure 5A,B), P/L ratios of 1/50 and 1/20 were compared. The ^31^P-NMR spectra indicate that at high concentration (P/L = 1/20, Figure 5B), the peptide strongly perturbs the membrane, as the degree of lipid orientation is massively decreased, with less than half of the lipids being arranged as well-oriented bilayers and the remainder contributing to an unoriented morphology. In POPC/POPG (Figure 5C,D), the lipid orientation at 1/20 was also very poor; hence only the spectra at 1/100 and 1/50 are shown. In DMPC/lyso-MPC (Figure 5E,F), the sample orientation was better, and even at a high peptide concentration of P/L = 1/20, the sample orientation was still quite good (in these spectra, there is a distinct peak from oriented lyso-lipids at approximately 20 ppm). In all lipid systems, a single ^15^N-NMR peak was found close to 100 ppm, with no indication of any change in peptide orientation between the lipid systems (e.g., as a function of spontaneous curvature) or as a function of peptide concentration. The position of the ^15^N-NMR signal represents the alignment of the chemical shift anisotropy tensor (CSA, with its main axis roughly aligned along the ^15^N-^1^H bond vector) of the labeled peptide bond in the oriented membrane sample. The observed ^15^N chemical shift of 100 ppm is fully consistent with β-strands that are aligned either flat on the membrane surface (as in Figure 1A–C) or with peptides aligned in a transmembrane orientation forming some kind of β-barrel type structure (as in Figure 1D,E). On the other hand, these ^15^N-NMR data do not support model F, in which the peptides form some kind of β-helix (Figure 1F), which can thus be ruled out with confidence.

While ^15^N-NMR has allowed us to examine the peptide backbone, oriented ^19^F-NMR experiments will next provide complementary information on the alignment of the peptide side chains, namely on the direction of the C_α_-C_β_ bond vector. The rigid amino acid CF_3_-Bpg is used as a ^19^F-label, because its side chain (i.e., the CF_3_-bond vector) extends perpendicularly to the plane of a β-sheet/-strand. This means that the relevant dipolar vector of the CF_3_-group is aligned essentially orthogonally to the principal axis of the ^15^N CSA tensor. It should therefore be possible to distinguish the different peptide architectures of models discussed in the introduction in which the peptide backbone lies flat on the membrane surface. When the C-CF_3_ bond vector of an immobilized peptide points straight down into the membrane (Figure 1C), i.e., parallel to the bilayer normal, this will produce a dipolar splitting of approximately +15 kHz. On the other hand, in a flipped peptide, the C-CF_3_ bond vector is oriented 90° to the membrane normal, giving a splitting of approximately −7.5 kHz, which would be expected for a β-barrel (Figure 1D), a toroidal wormhole (Figure 1E) or a β-helix (Figure 1F). Besides these orientational aspects, the ^19^F-NMR analysis also provides important information on peptide mobility, which will help to discriminate highly dynamic monomeric peptides (as in Figure 1A,B) from immobilized oligomeric assemblies (Figure 1C) and detect uniaxial rotational averaging around the membrane normal (as expected in the discrete pore models of Figure 1D–F).

**Figure 6:**
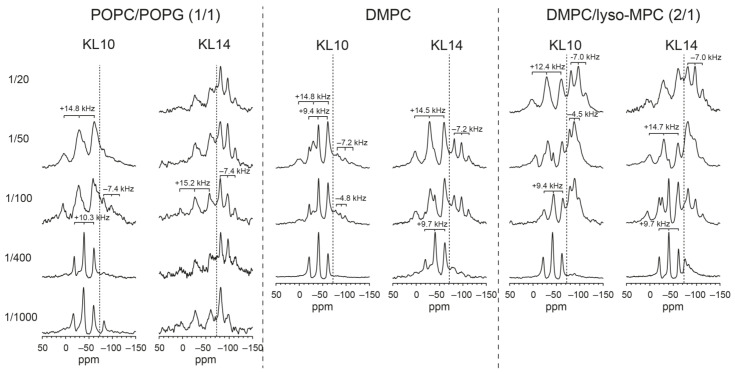
Solid-state ^19^F-NMR spectra of KL10-^19^F and KL14-^19^F (labeled with a single CF_3_-group at position 6) in macroscopically oriented membrane samples aligned as above. Dotted lines indicate the isotropic chemical shift (−72 ppm). Triplets arise from the dipolar coupling between the ^19^F nuclei within the CF_3_-group, and the splitting depends on the orientation of the C-CF_3_ bond with regard to the membrane normal. Triplets to the left and right of the isotropic chemical shift have positive and negative signs of dipolar coupling, respectively. Three lipid systems were used, as denoted above each column, and several P/L ratios, as indicated on the left of each row of spectra (P/L values are for KL14, and the same peptide-to-lipid mass ratios were used for KL10).

^19^F-NMR experiments were performed on ^19^F-labeled KL10 and KL14 in the same lipid systems as used for the ^15^N-NMR analysis, and spectra are shown in Figure 6. Basically, two different splittings were observed. KL10 at low peptide concentrations of P/L = 1/1000 and 1/400 gave a splitting of 9–10 kHz, which at higher P/L increased to 12–15 kHz. In POPC/POPG, the larger splitting dominated already at P/L = 1/100, but in DMPC and DMPC/lyso-MPC, the smaller splitting was still observed at 1/50, co-existing with the larger splitting. In DMPC and DMPC/lyso-MPC at P/L = 1/50 and 1/100, a smaller negative splitting of −4.5 kHz was observed, which probably originates from non-oriented parts of the sample, where fast peptide motions lead to an averaging of the smaller 9 kHz splitting by a factor of −1/2. At P/L = 1/20 in DMPC/lyso-MPC, a splitting of about −7 kHz was seen, which indicates non-oriented immobilized peptides (a so-called “powder” signal). For KL14, the larger splitting is more dominant. In POPC/POPG, only a large splitting of 15 kHz is observed even at P/L = 1/1000. In DMPC and DMPC/lyso-MPC, the large splitting dominates at 1/100, though at 1/400, a smaller splitting of approximately 10 kHz is observed. At a high P/L, a powder splitting of −7.4 kHz was observed in all lipid systems. These ^19^F-NMR results indicate that the KL10 and KL14 peptide backbones are aligned flat on the membrane surface, i.e., compatible with models A/B/C illustrated in Figure 1. This will be explained in detail in the discussion section below.

## 4. Discussion

**Figure 7:**
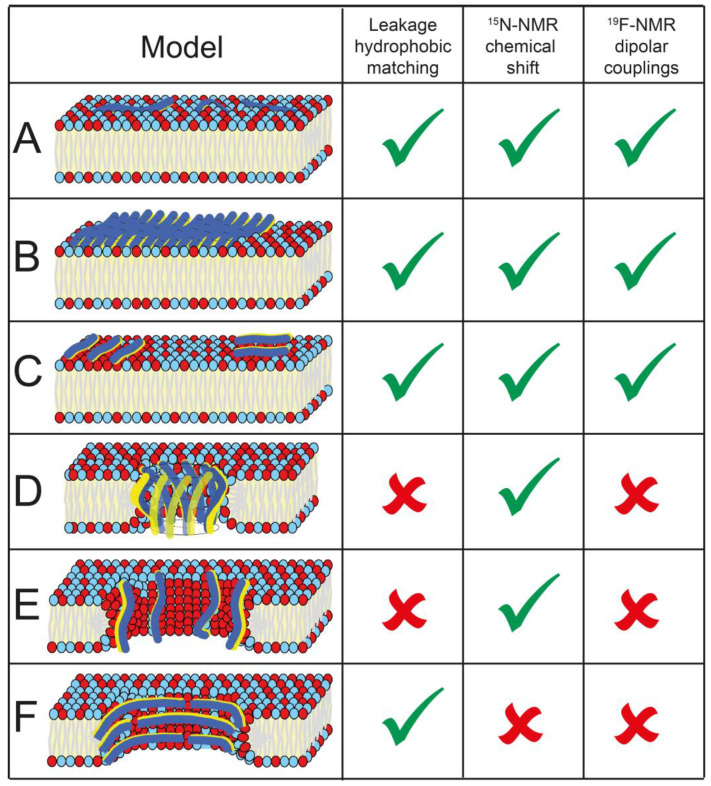
Compatibility of the different hypothetical models **A**–**F** from Figure 1 with our experimental data. A green tick (✓) indicates that the model is consistent with the data, while a red cross (✕) means that the model has to be rejected due to inconsistency with the data.

In this study, we have systematically explored how the length of amphipathic β-stranded peptides affects their membranolytic activity. KL model peptides with lengths from 6 to 26 amino acids were constructed from simple leucine-lysine repeats. We will first discuss and compare the activity of these different peptides against bacteria, erythrocytes and lipid vesicles and then relate these general activities with the structural data from CD and SSNMR spectroscopy. This way, the different architectures of models A–F illustrated in Figure 1 can be differentiated and discriminated for these novel KL-type peptides in order to come up with a mechanistic interpretation of antimicrobial action. Figure 7 shows an overview of the models and how well they fit with our experimental data.

The activity of KL peptides against four strains of bacteria shows a clear length dependence, with maximum antimicrobial activity for peptides with 9–15 amino acids (Table 2). Shorter and longer peptides are less active. On the other hand, the hemolytic activity increases steadily with length; peptides with 11 or more amino acids are strongly hemolytic, and there is clearly no reduction in activity for longer peptides (except at high peptide concentrations, where aggregation probably occurs). This finding means that the KL peptides have an “optimum” length of 9–11 amino acids, where they have both good antimicrobial activity and low hemolytic side effects. Shorter peptides are not active at all, and longer peptides have both lower antimicrobial activity and much higher hemolytic side effects, making them less therapeutically promising. For vesicle leakage, we observe a similar length dependence as that for hemolysis, with low activity for peptides with 10 or fewer amino acids and high activity for longer peptides. The size-dependent leakage assay shows that large molecules can also leak out of vesicles, and long KL26 peptides can induce leakage of larger molecules than short KL14 peptides can (Figure 4C). However, from the results of dextran leakage, it is hard to conclude the exact size of the holes formed. The analysis above assumes dextrans to be spherical, but it has been shown that the larger dextrans can be represented as prolate ellipsoids with large axial ratios (for example, the axial ratio for a dextran with a molecular mass of 33 kDa was found to be 16) [60]. Therefore, even those dextran molecules with a nominal hydrodynamic radius that is too large to fit in the 1.8–2.6 nm diameter pore formed by alamethicin [58,59] can leak out to some extent. By comparing KL14 and KL26 with alamethicin, we can see that KL14 forms lesions of a similar size as those of alamethicin, while KL26 forms somewhat larger lesions.

These results from the antimicrobial and hemolytic assays, as well as vesicle leakage tests, indicate that long KL peptides are intrinsically active when bound to the membrane. However, their strong tendency to aggregate almost instantaneously in aqueous solution leads to losses in “active” material that can reach the membrane. This outcome is especially significant in the case of the antimicrobial assay, where phosphate in the medium enhances aggregation but cannot be avoided.

Odd-numbered LK peptides are more hemolytic and less antimicrobial than the corresponding KL peptides. The odd-numbered LK peptides (sequence L[KL]_n_) have one more Leu than Lys, whereas the odd-numbered KL peptides (sequence [KL]_n_K) have one more Lys than Leu, so the balance between hydrophobicity and charge is shifted. Similar to what is known from the literature on α-helical peptides, the more hydrophobic odd-numbered LK peptides are also more hemolytic [52,53,61,62]. It appears that those odd-numbered LK peptides are therefore less useful as antimicrobial agents. We conclude that the most promising therapeutic candidates are KL10, LK10 and KL11, and of these, KL11 has the least undesired hemolytic side effects.

Why are the peptides with about 10 amino acids the best? This result must be explicable in terms of the structure and dynamics of the peptides, i.e., with their membranolytic mechanism, which we have investigated with biophysical methods. From CD spectroscopy in solution, we observed that in water, all peptides were unstructured (Figure 2A–C). In the presence of anionic POPC/POPG (1/1) vesicles, all peptides (except the very short KL6) formed β-stranded structures, with a CD minimum at ~217 nm and a maximum at ~197 nm (Figure 2D–F). A quantitative structure analysis was performed by deconvolution of the CD line shapes of KL10 in water at pH ≈ 10, as well as in 10 mM phosphate buffer (where β-sheets are also formed, but no turbidity artifacts impeded the analysis). In both cases, KL10 was found to form mostly antiparallel β-sheets [15]. For longer peptides, the intensity of the CD signals is significantly lower, which can be attributed to aggregation in solution, so that some of the material precipitates and does not contribute to the signal. We had previously shown that the aggregation tendency of the KL peptides increases steadily with length [15].

Oriented CD samples are prepared by co-dissolving peptides and lipids in organic solvents and drying them to a film on a solid support. Then, the sample is hydrated in a chamber with 97% relative humidity. Hence, there is only a limited amount of water available and no bulk water phase. In this case, the peptides cannot precipitate from solution but are forced to stay in/on the membrane. In POPC/POPG (1/1) lipid bilayers, all peptides gave line shapes indicative of β-sheets, but the shorter peptides (KL8, KL10, KL12) also showed signals from unstructured conformations, with a minimum at 195 nm (Figure 3A). It appears that these peptides have a lower propensity to form aggregated β-sheets in the membrane, which correlates with their high antimicrobial activity and low hemolytic activity.

In DMPC/lyso-MPC (2/1) lipids, all peptides formed β-sheets, and the short peptides did not contribute any unstructured signals. In unsaturated POPC/POPG bilayers, which have a negative spontaneous curvature, it has been observed that membrane-active peptides in general tend to reside ON/IN the membrane surface, i.e., with their amphiphilic faces parallel to the bilayer plane. In DMPC/lyso-MPC bilayers, however, which have a positive spontaneous curvature, the peptide backbones were seen to readily tilt into the membrane once a threshold concentration was reached [35,63,64]. These previous observations fit well with our OCD data, where the shorter peptides are less structured in POPC/POPG (residing on the surface) than in DMPC/lyso-MPC (able to penetrate more deeply into the bilayer).

When ^19^F-NMR spectroscopy was used to characterize KL10 and KL14 in oriented membranes, two distinct populations of peptides were observed (Figure 6). One population gives a ^19^F dipolar splitting of approximately +9 kHz, and the other one gives a splitting of approximately +15 kHz. In a previous study on KIGAKI peptides, an alternating amphiphilic β-strand forming sequence similar to that of the KL peptides, two sets of splittings had also been observed [14]. The smaller splitting (in that case, +7.5 kHz) could be assigned to more mobile, less structured peptides, while the larger splitting (of +15 to +17 kHz) was associated with peptides forming immobilized β-sheets. It seems that the KL peptides are also present in these two forms. Compared to KL10, the longer KL14 showed much more spectral intensity in the signal with the large splitting, which correlates well with its higher tendency to self-assemble as β-sheets, which is, of course, also concentration dependent. In POPC/POPG (1/1), KL10 gave the small +9 kHz splitting at low peptide concentrations of P/L = 1/1000 and 1/400, while the large +15 kHz splitting dominated at 1/100 and 1/50. KL14, on the other hand, gave the large splitting exclusively, even at a high dilution of 1/1000. In DMPC and DMPC/lyso-MPC, both peptides at 1/400 gave only the small +9 kHz splitting, while at 1/100, a larger splitting was also observed, which, for KL14, had a higher intensity than for KL10. The higher proportion of aggregated peptides in POPC/POPG may be related to the presence of charged phospholipids, as it was also shown previously that KL peptides aggregate strongly in the presence of phosphate ions [15].

It is very clear that there is a pronounced correlation between aggregation behavior and biological activity. Extremely short peptides (e.g., KL6) are completely inactive, as they neither bind to membranes nor do they aggregate with themselves. Excessively long peptides aggregate vigorously in solution before they can even reach the membrane surface. Therefore, the optimal length of peptides is approximately 10 amino acids. Slightly longer peptides have even better antimicrobial activity, but beyond KL14, this beneficial feature decreases again.

From a structural point of view, the optimal window for antibiotic activity may well be related to the mechanism of action, in analogy to helical pore-forming peptides. Alamethicin [65], magainins [66,67,68] and KIA peptides [51,54] have all been shown to form transmembrane pores in lipid bilayers (under suitable conditions). For KIA peptides, it was found that a certain minimum length was needed for membrane activity, i.e., the α-helix must be long enough to span the bilayer to be active [54]. For KIA peptides, a length of 21 amino acids was sufficient to kill the same bacteria that have been tested here [69]. This length corresponds to an α-helix of 31.5 Å, assuming a 1.5 Å increment per residue and a completely helical sequence. For an extended β-strand in an ideal β-sheet, the increment per residue is approximately 3.7 Å. Thus, a β-strand with 9 amino acids has a length (9 × 3.7 Å = 33.3 Å) similar to that of an α-helix with 21 amino acids (21 × 1.5 Å = 31.5 Å), which should thus be long enough to span the membrane. Such an extended β-strand would obviously have to be aligned in an upright transmembrane orientation and be accompanied by other strands to form a stable oligomeric pore, as illustrated in Figure 1D,E (Figure 7D,E). This hydrophobic mismatch hypothesis fits well with our bacterial MIC data above, showing that KL peptides shorter than 9 amino acids are not active. It can be argued that the highly antibiotic KL peptides with a length of up to 14 amino acids fit well across a lipid bilayer as a pore, provided that they can adjust their angle and tilt away slightly from the bilayer normal. Finally, KL peptides with a length of 15 amino acids or more are again less active, which would actually correspond in length to an α-helix with 37 amino acids. The longest α-helical KIA peptide that had been tested contained only 28 amino acids, so it is conceivable that such long KIA peptides would also show less activity. Alternatively, the reason could be the strong aggregation tendencies of long KL peptides, which are far stronger than those of any KIA peptides.

The hypothesis of KL peptides forming pores in a lipid bilayer in an upright, transmembrane orientation was tested in two ways. If KL peptides need a minimum length to span the membrane in order to be active (Figure 1D,E, Figure 7D,E), they should show different leakage thresholds in lipid vesicles that are composed of bilayers with different thicknesses, as had been observed for KIA peptides [54,70]. Fluorescent leakage experiments of KL peptides of different lengths were performed with thin membranes (DMoPC/DMoPG, hydrophobic thickness 19.2 Å [56]), intermediate membranes (POPC/POPG, hydrophobic thickness 27.1 Å [57] and 27.8 Å [71]), and thick membranes (DErPC/DErPG, hydrophobic thickness 34.4 Å [57]). Remarkably, in all cases, the same length dependence was found: low leakage for peptides up to 10 amino acids and high leakage for longer peptides (Figure 4B). This result clearly contradicts the hypothesis of membrane-spanning pores.

The second test of the pore hypothesis was performed using solid-state NMR. To obtain information about the orientation of α-helical peptides in membranes, ^15^N-NMR is a straightforward and very useful method. In macroscopically oriented samples, the chemical shift of the narrow peak from a single ^15^N-labeled amide group in the middle of the helical region provides the approximate tilt angle of the peptide [51,72]. For β-sheet-forming peptides such as the KL series, however, there is no such simple theory that can yield the corresponding information from ^15^N-labeled peptides. Nonetheless, the main reason for performing these experiments was the hope that a shift in the NMR signal would be observed for different KL lengths, at different concentrations, or in different bilayers, which could then be correlated with further ^19^F-NMR results. However, all experiments under all conditions showed more or less the same peak at approximately 100 ppm, so the ^15^N-NMR experiments were rather inconclusive. We can only exclude the unusual β-helical model in Figure 1F (Figure 7F) with some confidence because, in this case, a ^15^N-NMR chemical shift closer to 200 ppm would have been expected, as visualized in the corresponding vector diagrams. Note that the chemical shift of an oriented peptide depends on both CSA tensor orientation (and thereby segmental orientation or possibly even whole-body orientation of the peptide backbone) as well as dynamics (local segmental wobble and any larger-scale fluctuations). Therefore, in a potentially wobbly β-stranded peptide (monomer, oligomer, aggregate, or fibril), the position of the NMR signal cannot be translated into the peptide alignment, as it is routinely possible in the case of α-helical peptides.

^19^F-NMR experiments, finally, gave a clear result. If KL peptides form β-strands on the membrane surface as in Figure 1A–C (Figure 7A–C), then the CF_3_ label points down into the membrane, almost parallel to the bilayer normal (which is aligned with B_0_ of the NMR magnet). It should give a large splitting of about +15 kHz when the peptide is immobile (and slightly less in the case of some local or global motional averaging). If, on the other hand, peptides are placed upright in a transmembrane orientation as in Figure 1D,E (Figure 7D,E), the CF_3_ label should point almost perpendicular to the membrane normal and give half the splitting of −7.5 kHz when immobilized (or less in the case of dynamics). The ^19^F-NMR spectra in Figure 6 always gave large splittings corresponding to peptides oriented flat on the membrane surface. Additionally, in DMPC/lyso-MPC (2/1), in which it is generally very favorable for KIA peptides and other amphiphilic helices to assume a transmembrane orientation, no experimental evidence for an upright insertion was found for KL peptides. Therefore, we conclude that KL peptides do not form traditional pores in a transmembrane orientation, neither in the form of β-barrels consisting of H-bonded β-sheets (Figure 1D and Figure 7D), nor from monomers in a toroidal wormhole style (Figure 1E and Figure 7E). Additionally, β-helical pores with the peptide backbones oriented parallel to the membrane surface can also be excluded by the ^19^F-NMR data (Figure 1F and Figure 7F) since the CF_3_ groups would also, in this case, be oriented perpendicular to the membrane normal and give splittings of approximately −7.5 kHz, which is not observed.

It thus seems clear that KL peptides bind to the membrane surface and use some type of carpet mechanism to permeabilize the bilayer and induce leakage, forming defects of a limited size. Monomeric, flexible β-strands as in Figure 1A (Figure 7A) will obviously prevail at low peptide concentration, especially for short KL peptides with a low tendency to aggregate. Longer KL peptides form larger aggregates with less mobility. As seen above, there is also a lipid dependence, with, for example, more immobilized peptides seen in POPC/POPG than in DMPC/lyso-MPC. Aggregation into amyloid-like fibrils consisting of H-bonded β-sheets as in Figure 1B (Figure 7B) cannot be discriminated, at this point in time, from the kind of lateral self-assembly depicted in Figure 1C (Figure 7C), where the cationic peptides are segregated in some immobilized phase that is enriched with anionic lipids. Future solid-state NMR techniques might be able to provide more information about the detailed aggregation behavior.

## 5. Conclusions

Membrane-active antimicrobial peptides are typically cationic and amphipathic. Here, we have shown that very simple KL (and LK) model peptides, forming amphipathic β-strands, can have high antimicrobial activity and low hemolytic side effects at the same time. However, these KL peptides are therapeutically promising only with a length of 10 or 11 amino acids. Shorter KL peptides actually bind to the membrane [15], but they are not membranolytic, most likely because they do not form sufficiently large aggregates that are needed to permeabilize the lipid bilayer. Longer peptides, on the other hand, are extremely hemolytic. These long peptides aggregate vigorously already in solution, and therefore, few of them reach the membrane, especially in the antimicrobial MIC assay where phosphate ions are present in the buffer. Nonetheless, once the long peptides are bound to the membrane, they are as active as the intermediate peptides (on a mass-to-mass ratio). For any therapeutic or biotechnological applications, KL peptides should thus be long enough to be able to form aggregates in the membrane but be short enough to have some flexibility and not pre-aggregate in solution.

This conclusion indicates that the mechanism of action observed in bacteria and in red blood cells involves membrane permeabilization. Using ^19^F-NMR spectroscopy, we have shown that the KL peptides do not form pores in a transmembrane orientation, despite the intriguing length-dependent optimum in antimicrobial activity. Vesicle leakage data using different membrane thicknesses also indicate that these β-stranded peptides, unlike many α-helical antimicrobial peptides, do not have to span the membrane to form some sort of pore to be active. Instead, a carpet mechanism seems to be operating here, where the peptides transiently accumulate in the outer leaflet and cause such an imbalance in lateral pressure that the lipid bilayer forms local lesions. Remarkably, our size-dependent leakage experiments have shown that the diameter of these lesions is, nonetheless, neither indefinitely large nor small but rather comparable to the typical pore diameters of alamethicin and other α-helical antibiotics.

## Figures and Tables

**Table 1:** Synthesized peptides used in this study. The net charge and the theoretical length of peptides in an ideal β-strand conformation are shown.

Peptide	Sequence	Net Charge	Length ^a^/Å
KL6	KLKLKL-NH_2_	+4	22.2
KL8	KLKLKLKL-NH_2_	+5	29.6
KL9	KLKLKLKLK-NH_2_	+6	33.3
LK9	LKLKLKLKL-NH_2_	+5	33.3
KL10	KLKLKLKLKL-NH_2_	+6	37.0
LK10	LKLKLKLKLK-NH_2_	+6	37.0
KL11	KLKLKLKLKLK-NH_2_	+7	40.7
LK11	LKLKLKLKLKL-NH_2_	+6	40.7
KL12	KLKLKLKLKLKL-NH_2_	+7	44.4
KL13	KLKLKLKLKLKLK-NH_2_	+8	48.1
LK13	LKLKLKLKLKLKL-NH_2_	+7	48.1
KL14	KLKLKLKLKLKLKL-NH_2_	+8	51.8
KL15	KLKLKLKLKLKLKLK-NH_2_	+9	55.5
LK15	LKLKLKLKLKLKLKL-NH_2_	+8	55.5
KL16	KLKLKLKLKLKLKLKL-NH_2_	+9	59.2
KL18	KLKLKLKLKLKLKLKLKL-NH_2_	+10	66.6
KL20	KLKLKLKLKLKLKLKLKLKL-NH_2_	+11	74.0
KL22	KLKLKLKLKLKLKLKLKLKLKL-NH_2_	+12	81.4
KL24	KLKLKLKLKLKLKLKLKLKLKLKL-NH_2_	+13	88.8
KL26	KLKLKLKLKLKLKLKLKLKLKLKLKL-NH_2_	+14	96.2
KL10-^15^N	KLKLK-(^15^N-Leu)-KLKL-NH_2_	+6	37.0
KL14-^15^N	KLKLK-(^15^N-Leu)-KLKLKLKL-NH_2_	+8	51.8
KL10-^19^F	KLKLK-(CF_3_-Bpg)-KLKL-NH_2_	+6	37.0
KL14-^19^F	KLKLK-(CF_3_-Bpg)-KLKLKLKL-NH_2_	+8	51.8

^a^ Approximate length, assuming 3.7 Å per residue for an ideal β-sheet.

**Table 2:** MIC values for KL peptides against four bacterial strains and HC_50_ data (µg/mL).

Peptide	Gram-Negative		Gram-Positive		Hemolysis ^a^
	*E. coli*	*E. helveticus*	*B. subtilis*	*S. xylosus*	HC_50_
KL6	>256	128	>256	256	>256
KL8	64	16	16	8	>256
KL9	32	8	16	2	>256
LK9	16	4	4	2	145
KL10 ^b^	4	2	2	2	47
LK10 ^b^	8	2	4	1	256
KL11 ^b^	4	2	4	1	>256
LK11	2	1	2	1	3
KL12	2	2	2	2	8
KL13	2	1	2	1	9
LK13	8	4	8	4	2
KL14	8	4	4	4	1
KL15	8	2	4	2	2
LK15	64	16	16	16	<1
KL16	32	8	16	8	2
KL18	32	16	32	8	1
KL20	32	32	32	16	<1
KL22	32	32	32	16	<1
KL24	32	16	32	8	<1
KL26	64	16	32	16	<1

^a^ Note that HC_50_ values are obtained from concentration-dependent curves by interpolation (Supplementary Figure S2); therefore, these values do not correspond to powers of 2^n^ such as the MIC values. ^b^ The peptide candidates with the most promising therapeutic profile (low MIC value and high HC_50_ value) are highlighted in grey.

## Data Availability

The experimental raw data are available from the authors upon request.

## Supplementary Materials

The following Supplementary Material can be downloaded at https://www.mdpi.com/article/10.3390/biomedicines10092071/s1, Method descriptions with additional details; Table S1: Size of the FITC-dextrans according to dynamic light scattering measurements, assuming spherical particles. The size of carboxyfluorescein (CF) is taken from [13]; Table S2: Hemolysis (in %) at different KL peptide concentrations (µg/mL). Values are compared with the values after addition of triton-X, which leads to 100% hemolysis. For KL14, KL16 and longer peptides the hemolysis is no longer increasing continuously with peptide concentration, probably due to aggregation; Figure S1: Results of size determination of FITC-dextrans by dynamic light scattering; Figure S2: LC-MS chromatograms showing absorbance at 220 nm (A,C,E,G) and mass spectra (B,D,F,H) of selected peptides used in this study. In all cases there is a single peak in the chromato-gram and the expected mass is found for each peptide. (A,B) KL10, (C,D) KL10-19F, (E,F) KL14, (G,H) KL18; Figure S3: Circular dichroism spectra of KL and LK peptides. (A–C) CD spectra in pure water, at pH ≈ 9–10. The peptide concentration was 0.1 mg/mL. (D–F) CD spectra in the presence of DMPC SUVs at pH ≈ 9–10. The lipid concentration was 1 mg/mL (1.5 mM) and the peptide concentration for KL14 was 0.15 mg/mL (75 μM) resulting in a peptide-to-lipid (P/L) molar ratio of 1/20. The other samples were prepared in the same peptide-to-lipid mass-to-mass ratio to keep the charge ratio constant; Figure S4: Hemolytic activity of KL and LK peptides. (A) Peptides with length 6–11 amino acids. (B) Peptides with length 12–15 amino acids. (C) Peptides with length 16–26 amino acids. In general, longer peptides are more active. For very long peptides, activity goes down at higher concentration, probably due to peptide aggregation. The dotted line in each panel indicates 50% hemolysis, and the HC50 value is determined from the crossing of the hemolysis curves with this line. References [6,14,15,26,27,28,29,30,31,32,33,34,35,36,37,73,74,75,76,77,78,79,80,81] are cited in the Supplementary Materials.

## References

[1] Pinheiro da Silva F., Machado M.C. (2012). Antimicrobial peptides: Clinical relevance and therapeutic implications. Peptides.

[2] Wimley W.C., Hristova K. (2011). Antimicrobial peptides: Successes, challenges and unanswered questions. J. Membr. Biol..

[3] Arias C.A., Murray B.E. (2009). Antibiotic-resistant bugs in the 21st century—A clinical super-challenge. N. Engl. J. Med..

[4] Brogden K.A. (2005). Antimicrobial peptides: Pore formers or metabolic inhibitors in bacteria?. Nat. Rev. Microbiol..

[5] Boman H.G. (2003). Antibacterial peptides: Basic facts and emerging concepts. J. Int. Med..

[6] Bürck J., Wadhwani P., Fanghänel S., Ulrich A.S. (2016). Oriented circular dichroism: A method to characterize membrane-active peptides in oriented lipid bilayers. Acc. Chem. Res..

[7] Strandberg E., Ulrich A.S. (2004). NMR methods for studying membrane-active antimicrobial peptides. Concepts Magn. Reson. A.

[8] Strandberg E., Ulrich A.S., Webb G. (2017). Solid-state ^19^F-NMR analysis of peptides in oriented biomembranes. Modern Magnetic Resonance.

[9] Strandberg E., Ulrich A.S., Webb G.A. (2017). Solid-state NMR for studying peptide structures and peptide-lipid interactions in membranes. Modern Magnetic Resonance.

[10] Wadhwani P., Strandberg E., Ojima I. (2009). Structure analysis of membrane-active peptides using ^19^F-labeled amino acids and solid-state NMR. Fluorine in Medicinal Chemistry and Chemical Biology.

[11] Strandberg E., Ulrich A.S. (2020). Flow charts for the systematic solid-state ^19^F/^2^H-NMR structure analysis of membrane-bound peptides. Annu. Rep. NMR Spectrosc..

[12] Blazyk J., Wiegand R., Klein J., Hammer J., Epand R.M., Epand R.F., Maloy W.L., Kari U.P. (2001). A novel linear amphipathic β-sheet cationic antimicrobial peptide with enhanced selectivity for bacterial lipids. J. Biol. Chem..

[13] Wadhwani P., Reichert J., Strandberg E., Bürck J., Misiewicz J., Afonin S., Heidenreich N., Fanghänel S., Mykhailiuk P.K., Komarov I.V. (2013). Stereochemical effects on the aggregation and biological properties of the fibril-forming peptide [KIGAKI]_3_ in membranes. Phys. Chem. Chem. Phys..

[14] Wadhwani P., Strandberg E., Heidenreich N., Bürck J., Fanghänel S., Ulrich A.S. (2012). Self-assembly of flexible β-strands into immobile amyloid-like β-sheets in membranes as revealed by solid-state ^19^F NMR. J. Am. Chem. Soc..

[15] Strandberg E., Schweigardt F., Wadhwani P., Bürck J., Reichert J., Cravo H.L.P., Burger L., Ulrich A.S. (2020). Phosphate-dependent aggregation of [KL]_n_ peptides affects their membranolytic activity. Sci. Rep..

[16] Grage S.L., Afonin S., Ieronimo M., Berditsch M., Wadhwani P., Ulrich A.S. (2022). Probing and manipulating the lateral pressure profile in lipid bilayers using membrane-active peptides: A solid-state ^19^F NMR study. Int. J. Mol. Sci..

[17] Silvius J.R., Gagné J. (1984). Calcium-induced fusion and lateral phase separations in phosphatidylcholine-phosphatidylserine vesicles–Correlation by calorimetric and fusion measurements. Biochemistry.

[18] Serra-Batiste M., Ninot-Pedrosa M., Bayoumi M., Gairi M., Maglia G., Carulla N. (2016). Aβ42 assembles into specific β-barrel pore-forming oligomers in membrane-mimicking environments. Proc. Natl. Acad. Sci. USA.

[19] Jang H., Arce F.T., Ramachandran S., Capone R., Lal R., Nussinov R. (2010). β-Barrel topology of Alzheimer’s β-amyloid ion channels. J. Mol. Biol..

[20] Kandel N., Matos J.O., Tatulian S.A. (2019). Structure of amyloid β(25-35) in lipid environment and cholesterol-dependent membrane pore formation. Sci. Rep..

[21] Mani R., Cady S.D., Tang M., Waring A.J., Lehrer R.I., Hong M. (2006). Membrane-dependent oligomeric structure and pore formation of a β-hairpin antimicrobial peptide in lipid bilayers from solid-state NMR. Proc. Natl. Acad. Sci. USA.

[22] Ketchem R.R., Hu W., Cross T.A. (1993). High-resolution conformation of gramicidin A in a lipid bilayer by solid-state NMR. Science.

[23] Carpino L.A., Han G.Y. (1972). 9-Fluorenylmethoxycarbonyl amino-protecting group. J. Org. Chem..

[24] Afonin S., Mikhailiuk P.K., Komarov I.V., Ulrich A.S. (2007). Evaluating the amino acid CF_3_-bicyclopentylglycine as a new label for solid-state ^19^F-NMR structure analysis of membrane-bound peptides. J. Pept. Sci..

[25] Ruden S., Hilpert K., Berditsch M., Wadhwani P., Ulrich A.S. (2009). Synergistic interaction between silver nanoparticles and membrane-permeabilizing antimicrobial peptides. Antimicrob. Agents Chemother..

[26] Strandberg E., Tiltak D., Ieronimo M., Kanithasen N., Wadhwani P., Ulrich A.S. (2007). Influence of C-terminal amidation on the antimicrobial and hemolytic activities of cationic α-helical peptides. Pure Appl. Chem..

[27] Duzgunes N., Wilschut J. (1993). Fusion assays monitoring intermixing of aqueous contents. Methods Enzymol..

[28] Steinbrecher T., Prock S., Reichert J., Wadhwani P., Zimpfer B., Bürck J., Berditsch M., Elstner M., Ulrich A.S. (2012). Peptide-lipid interactions of the stress-response peptide TisB that induces bacterial persistence. Biophys. J..

[29] Ellens H., Bentz J., Szoka F.C. (1985). H^+^- and Ca^2+^-induced fusion and destabilization of liposomes. Biochemistry.

[30] Ladokhin A.S., Selsted M.E., White S.H. (1997). Sizing membrane pores in lipid vesicles by leakage of co-encapsulated markers: Pore formation by melittin. Biophys. J..

[31] Stutzin A. (1986). A fluorescence assay for monitoring and analyzing fusion of biological membrane-vesicles in vitro. FEBS Lett..

[32] Grage S.L., Strandberg E., Wadhwani P., Esteban-Martin S., Salgado J., Ulrich A.S. (2012). Comparative analysis of the orientation of transmembrane peptides using solid-state ^2^H- and ^15^N-NMR: Mobility matters. Eur. Biophys. J..

[33] Müller S.D., De Angelis A.A., Walther T.H., Grage S.L., Lange C., Opella S.J., Ulrich A.S. (2007). Structural characterization of the pore forming protein TatA_d_ of the twin-arginine translocase in membranes by solid-state ^15^N-NMR. Biochim. Biophys. Acta.

[34] Glaser R.W., Sachse C., Dürr U.H.N., Afonin S., Wadhwani P., Strandberg E., Ulrich A.S. (2005). Concentration-dependent realignment of the antimicrobial peptide PGLa in lipid membranes observed by solid-state ^19^F-NMR. Biophys. J..

[35] Strandberg E., Zerweck J., Wadhwani P., Ulrich A.S. (2013). Synergistic insertion of antimicrobial magainin-family peptides in membranes depends on the lipid spontaneous curvature. Biophys. J..

[36] Heinzmann R., Grage S.L., Schalck C., Bürck J., Banoczi Z., Toke O., Ulrich A.S. (2011). A kinked antimicrobial peptide from *Bombina maxima*. II. Behavior in phospholipid bilayers. Eur. Biophys. J..

[37] Walther T.H., Grage S.L., Roth N., Ulrich A.S. (2010). Membrane alignment of the pore-forming component TatA_d_ of the twin-arginine translocase from *Bacillus subtilis* resolved by solid-state NMR spectroscopy. J. Am. Chem. Soc..

[38] Cullis P.R., de Kruijff B. (1979). Lipid polymorphism and the functional roles of lipids in biological membranes. Biochim. Biophys. Acta.

[39] Wadhwani P., Tremouilhac P., Strandberg E., Afonin S., Grage S.L., Ieronimo M., Berditsch M., Ulrich A.S., Soloshonok V.A., Mikami K., Yamazaki T., Welch J.T., Honek J.F. (2007). Using fluorinated amino acids for structure analysis of membrane-active peptides by solid-state ^19^F-NMR. Current Fluoroorganic Chemistry: New Synthetic Directions, Technologies, Materials, and Biological Applications.

[40] Teng Q., Cross T.A. (1989). The *in situ* determination of the ^15^N chemical-shift tensor orientation in a polypeptide. J. Magn. Reson..

[41] Bechinger B., Zasloff M., Opella S.J. (1993). Structure and orientation of the antibiotic peptide magainin in membranes by solid-state nuclear magnetic resonance spectroscopy. Protein Sci..

[42] Glaser R.W., Sachse C., Dürr U.H.N., Wadhwani P., Ulrich A.S. (2004). Orientation of the antimicrobial peptide PGLa in lipid membranes determined from ^19^F-NMR dipolar couplings of 4-CF_3_-phenylglycine labels. J. Magn. Reson..

[43] Dürr U.H.N., Grage S.L., Witter R., Ulrich A.S. (2008). Solid state ^19^F NMR parameters of fluorine-labeled amino acids. Part I: Aromatic substituents. J. Magn. Reson..

[44] Grage S.L., Durr U.H., Afonin S., Mikhailiuk P.K., Komarov I.V., Ulrich A.S. (2008). Solid state ^19^F NMR parameters of fluorine-labeled amino acids. Part II: Aliphatic substituents. J. Magn. Reson..

[45] Miles A.J., Wallace B.A. (2016). Circular dichroism spectroscopy of membrane proteins. Chem. Soc. Rev..

[46] Bürck J., Roth S., Wadhwani P., Afonin S., Kanithasen N., Strandberg E., Ulrich A.S. (2008). Conformation and membrane orientation of amphiphilic helical peptides by oriented circular dichroism. Biophys. J..

[47] Huang H.W., Wu Y. (1991). Lipid-alamethicin interactions influence alamethicin orientation. Biophys. J..

[48] Bazzi M.D., Woody R.W. (1987). Interaction of amphipathic polypeptides with phospholipids: Characterization of conformations and the CD of oriented β-sheets. Biopolymers.

[49] Woody R.W. (1993). The circular dichroism of oriented β sheets: Theoretical predictions. Tetrahedron Asymmetry.

[50] Strandberg E., Bentz D., Wadhwani P., Ulrich A.S. (2020). Chiral supramolecular architecture of stable transmembrane pores formed by an α-helical antibiotic peptide in the presence of lyso-lipids. Sci. Rep..

[51] Grau-Campistany A., Strandberg E., Wadhwani P., Rabanal F., Ulrich A.S. (2016). Extending the hydrophobic mismatch concept to amphiphilic membranolytic peptides. J. Phys. Chem. Lett..

[52] Strandberg E., Kanithasen N., Bürck J., Wadhwani P., Tiltak D., Zwernemann O., Ulrich A.S. (2008). Solid state NMR analysis comparing the designer-made antibiotic MSI-103 with its parent peptide PGLa in lipid bilayers. Biochemistry.

[53] Dathe M., Wieprecht T., Nikolenko H., Handel L., Maloy W.L., MacDonald D.L., Beyermann M., Bienert M. (1997). Hydrophobicity, hydrophobic moment and angle subtended by charged residues modulate antibacterial and haemolytic activity of amphipathic helical peptides. FEBS Lett..

[54] Grau-Campistany A., Strandberg E., Wadhwani P., Reichert J., Bürck J., Rabanal F., Ulrich A.S. (2015). Hydrophobic mismatch demonstrated for membranolytic peptides, and their use as molecular rulers to measure bilayer thickness in native cells. Sci. Rep..

[55] Epand R.F., Savage P.B., Epand R.M. (2007). Bacterial lipid composition and the antimicrobial efficacy of cationic steroid compounds (Ceragenins). Biochim. Biophys. Acta.

[56] Marsh D. (2008). Energetics of hydrophobic matching in lipid-protein interactions. Biophys. J..

[57] Kucerka N., Tristram-Nagle S., Nagle J.F. (2006). Structure of fully hydrated fluid phase lipid bilayers with monounsaturated chains. J. Membr. Biol..

[58] He K., Ludtke S.J., Worcester D.L., Huang H.W. (1996). Neutron scattering in the plane of membranes: Structure of alamethicin pores. Biophys. J..

[59] Qian S., Wang W.C., Yang L., Huang H.W. (2008). Structure of the alamethicin pore reconstructed by x-ray diffraction analysis. Biophys. J..

[60] Bohrer M.P., Deen W.M., Robertson C.R., Troy J.L., Brenner B.M. (1979). Influence of molecular configuration on the passage of macromolecules across the glomerular capillary wall. J. Gen. Physiol..

[61] Chen Y.X., Mant C.T., Farmer S.W., Hancock R.E.W., Vasil M.L., Hodges R.S. (2005). Rational design of alpha-helical antimicrobial peptides with enhanced activities and specificity/therapeutic index. J. Biol. Chem..

[62] Zamora-Carreras H., Strandberg E., Mühlhäuser P., Bürck J., Wadhwani P., Jiménez M.Á., Bruix M., Ulrich A.S. (2016). Alanine scan and ^2^H NMR analysis of the membrane-active peptide BP100 point to a distinct carpet mechanism of action. Biochim. Biophys. Acta.

[63] Strandberg E., Ulrich A.S. (2015). AMPs and OMPs: Is the folding and bilayer insertion of β-stranded outer membrane proteins governed by the same biophysical principles as for α-helical antimicrobial peptides?. Biochim. Biophys. Acta.

[64] Strandberg E., Tiltak D., Ehni S., Wadhwani P., Ulrich A.S. (2012). Lipid shape is a key factor for membrane interactions of amphipathic helical peptides. Biochim. Biophys. Acta.

[65] Leitgeb B., Szekeres A., Manczinger L., Vagvolgyi C., Kredics L. (2007). The history of alamethicin: A review of the most extensively studied peptaibol. Chem. Biodivers..

[66] Matsuzaki K. (1998). Magainins as paradigm for the mode of action of pore forming polypeptides. Biochim. Biophys. Acta.

[67] Yang L., Weiss T.M., Lehrer R.I., Huang H.W. (2000). Crystallization of antimicrobial pores in membranes: Magainin and protegrin. Biophys. J..

[68] Ludtke S.J., He K., Heller W.T., Harroun T.A., Yang L., Huang H.W. (1996). Membrane pores induced by magainin. Biochemistry.

[69] Strandberg E., Bentz D., Wadhwani P., Bürck J., Ulrich A.S. (2020). Terminal charges modulate the pore forming activity of cationic amphipathic helices. BBA-Biomembranes.

[70] Gagnon M.C., Strandberg E., Grau-Campistany A., Wadhwani P., Reichert J., Bürck J., Rabanal F., Auger M., Paquin J.F., Ulrich A.S. (2017). Influence of the length and charge on the activity of α-helical amphipathic antimicrobial peptides. Biochemistry.

[71] Pan J., Heberle F.A., Tristram-Nagle S., Szymanski M., Koepfinger M., Katsaras J., Kucerka N. (2012). Molecular structures of fluid phase phosphatidylglycerol bilayers as determined by small angle neutron and X-ray scattering. Biochim. Biophys. Acta.

[72] Bechinger B., Gierasch L.M., Montal M., Zasloff M., Opella S.J. (1996). Orientations of helical peptides in membrane bilayers by solid state NMR spectroscopy. Solid State Nucl. Magn. Reson..

[73] Rance M., Byrd R.A. (1983). Obtaining high-fidelity spin-1/2 powder spectra in anisotropic media–Phase-cycled Hahn echo spectroscopy. J. Magn. Reson..

[74] Levitt M.H., Suter D., Ernst R.R. (1986). Spin dynamics and thermodynamics in solid-state NMR cross polarization. J. Chem. Phys..

[75] Fung B.M., Khitrin A.K., Ermolaev K. (2000). An improved broadband decoupling sequence for liquid crystals and solids. J. Magn. Reson..

[76] Zhang S., Wu X.L., Mehring M. (1990). Elimination of ringing effects in multiple-pulse sequences. Chem. Phys. Lett..

[77] Bennett A.E., Rienstra C.M., Auger M., Lakshmi K.V., Griffin R.G. (1995). Heteronuclear decoupling in rotating solids. J. Chem. Phys..

[78] Reichert J., Grasnick D., Afonin S., Bürck J., Wadhwani P., Ulrich A.S. (2007). A critical evaluation of the conformational requirements of fusogenic peptides in membranes. Eur. Biophys. J..

[79] Sigma-Aldrich Fluorescein Isothiocyanate-Dextran. https://www.sigmaaldrich.com/technical-documents/protocols/biology/fluorescein-isothiocyanate-dextran.html#ref.

[80] Wooten M.K.C., Koganti V.R., Zhou S.S., Rankin S.E., Knutson B.L. (2016). Synthesis and nanofiltration membrane performance of oriented mesoporous silica thin films on macroporous supports. ACS Appl. Mater. Interfaces.

[81] Mayer L.D., Hope M.J., Cullis P.R. (1986). Vesicles of variable sizes produced by a rapid extrusion procedure. Biochim. Biophys. Acta.

