# Supplementary material for: Membranolytic Mechanism of Amphiphilic Antimicrobial β-Stranded [KL]n Peptides
Source: Biomedicines. 2022 Aug 24;10(9):2071. doi: 10.3390/biomedicines10092071 (PMC9495826; doi:10.3390/biomedicines10092071)
Supplement: Supplementary file 1 [file biomedicines-10-02071-s001.zip › biomedicines-1860084-supplementary.pdf]

# Supplementary Materials

# Membranolytic mechanism of amphiphilic antimicrobial β-stranded $[KL]_n$ peptides

**Fabian Schweigardt[1], Erik Strandberg[2,*], Parvesh Wadhwani[2], Johannes Reichert[2], Jochen Bürck[2], Haroldo L. P. Cravo[3], Luisa Burger[2], and Anne S. Ulrich[1,2,*]**

[1] Karlsruhe Institute of Technology (KIT), Institute of Organic Chemistry, Fritz-Haber-Weg 6, 776131 Karlsruhe, Germany
[2] KIT, Institute of Biological Interfaces (IBG-2), P.O. Box 3640, 76021 Karlsruhe, Germany
[3] Laboratório de Biofísica Molecular, Universidade de São Paulo, 14040-901 Ribeirão Preto, SP, Brazil
* Correspondence: erik.strandberg@kit.edu (E.S.); anne.ulrich@kit.edu (A.S.U.)

## 1. Methods with additional details

### *1.1. Circular dichroism spectroscopy (CD)*

#### 1.1.1. Sample preparation

For CD measurements in liposomes, stock solutions of the lipids (5 mg/mL in $CHCl_3$ /MeOH 1/1 vol/vol) and of the lyophilized peptides (1 mg/mL in $H_2O$/MeOH 1/9 vol/vol) were prepared. The final KL samples for CD measurements in pure water were obtained by diluting aliquots of the peptide stock solutions with deionized water, resulting in a peptide concentration of 0.1 mg/mL (27 – 113 µM, depending on the molecular weight of the peptide). POPC/POPG (1/1 mol/mol) and DMPC were chosen as lipid systems for the CD measurements in liposomes. The lipid concentration in the sample was adjusted to 1 mg/mL to get an analyzable CD signal of the peptides. Merging of peptide and lipid mol-ecules on a molecular level without preceding aggregation of peptides was achieved by mixing suitable aliquots of the organic peptide and lipid stock solutions (co-solubiliza-tion). For KL14 a molar peptide-to-lipid ratio (P/L) of 1/20 was chosen, corresponding to a concentration of 65 µM in DMPC (or 75 µM in POPC/ POPG 1:1) for the peptide and 1.3 mM or 1.5 mM for the lipid, respectively. The same corresponding peptide-to-lipid mass-to-mass ratio was used for all other KL peptides in order to keep the charge ratio constant, resulting in differing molar P/L ratios. After mixing of the organic peptide/lipid solutions and subsequent evaporation of the organic solvents under $N_2$ gas flow, the residual pep-tide/lipid film was vacuum dried for 4 h and re-dissolved in $H_2O$. Then, the samples were vortexed for 5 min and freeze/thaw-cycled tenfold. In a final step, the solutions were treated in an ultrasonic bath (UTR 200, Hielscher, Germany) for 16 min to generate small unilamellar vesicles (SUVs), which were stored overnight at room temperature.

Before the measurement of one series of aqueous peptide solutions, the samples were kept at the slightly acidic pH (~5-6) produced by the residual HCl from HPLC purification in the lyophilized peptides. In a second series of aqueous peptide solutions, and also for the liposome dispersions, the pH was set to 9-10 by adding a small aliquot (2-3 µL) of 0.1 M NaOH to each sample. The pH of each sample was checked with indicator paper.

#### 1.1.2. Measurements

The CD measurements were conducted using a J-815 circular dichroism spectropolarimeter (Jasco, Groß-Umstadt, Germany). Spectra were recorded in the spectral range from 260-190 nm, at 0.1 or 0.5 nm intervals with a scanning speed of 10 nm/min and a spectral bandwidth of 1 nm as described earlier [1]. Samples were measured in a 1 mm quartz glass cuvette (110-QS, Hellma Analytics, Müllheim, Germany) at 30°C both for the aqueous peptide solutions as well as for the lipid dispersions. For each sample, three separate scans were recorded, averaged, and the average baseline of the corresponding sample matrix without peptide (i.e. pure water or the lipid vesicle dispersion in water) was subtracted. Afterwards, the spectra were smoothed, and the baseline was zeroed at 260 nm using the Jasco Spectra Analysis software. Finally, all spectra were converted to mean residue ellipticity (MRE) units to allow for an unbiased spectral comparison of the peptides with different backbone lengths.

### *1.2. Oriented CD*

To examine the membrane alignment and conformation of the KL peptides in different lipid systems, oriented CD (OCD) experiments were performed on a Jasco J-810 spectropolarimeter with an OCD cell built in-house [2]. The samples were prepared from peptide stock solutions in MeOH/$H_2O$ (9/1 vol/vol) with a concentration of 1 mg/mL, and lipid stock solutions in MeOH/$CHCl_3$ (1/1 vol/vol) with a concentration of 5 mg/mL. The chosen lipid systems were POPC/POPG (1/1 mol/mol) and DMPC/lyso-MPC (2/1 mol/mol). These components were mixed and vortexed in the desired P/L ratio. To keep the charge ratio between peptide and lipid constant, all samples were prepared in a constant mass-to-mass ratio using KL14 as the reference peptide for which the molar P/L ratio was calculated. The respective mixture was then deposited on a circular fused silica plate using a gastight syringe. For achieving comparable layer thicknesses, all material was spread onto a circular area with a diameter of 13.5 mm in the center of the plate. For optimizing the signal-to-noise ratio, the samples were calculated to contain a maximum of 200 μg lipids and 15 μg peptide in a volume of 30-50 μL solvent. After evaporation of the solvent, the plates were vacuum dried for 3 h to remove residual solvent, and afterwards installed in a cylindrical sample holder containing a second fused silica plate. This container was then placed in a custom-built hydration cell, which was sealed by the fused silica plates of the holder (the one of the holder plus the one containing the sample). In this inner compartment of the sample holder, the peptide/lipid film was hydrated overnight at 30°C via the gas phase, using a saturated $K_2SO_4$ solution contained in an adjacent cavity of the hydration cell, which resulted in a relative humidity of ~97%.

For the actual measurement, the sample holder was taken out of the hydration cell and fixed in the OCD measurement cell, which was mounted on a rotational stage inside the sample compartment of the spectropolarimeter. It was oriented perpendicular to the beam direction, which allowed automatic rotation around the beam axis without having to interfere manually between each measurement angle. Right before each measurement, the humidity and temperature inside the measurement cell was checked by an integrated sensor (SHT 75, Sensirion, Zurich, Switzerland). The spectra were then taken at wavelengths from 260-185 nm at 8 rotation angles (0°, 45°, 90°, …, 315°) with a scanning speed of 10 nm/min and a spectral bandwidth of 1 nm. Two spectral scans were performed at each rotation angle. These measurements at different rotation angles were done to avoid artefacts caused by linear dichroism. The final spectrum was obtained by averaging all spectra of all angles, and baseline zeroing them at 260 nm. In addition to the regular samples, lipid reference samples were measured, containing the same amount of lipid as the corresponding regular sample but no peptide. These were recorded in the same way as the regular samples and the signals subtracted from the respective peptide spectra.

*1.3 MIC (minimum inhibitory concentration) assay*

Antimicrobial activity was measured by a minimal inhibitory concentration (MIC) assay, carried out with Gram-positive *Bacillus subtilis* (DSM 347) and *Staphylococcus xylosus* (DSM 20287) and with Gram-negative *Escherichia coli* (DSM 1116) and *Enterobacter helveticus* (DSM 18390). Bacteria were grown in Müller-Hinton medium at 37°C overnight. Bacterial solutions with $OD_{550}$ = 0.2 was further diluted 1:100 for Gram-positive and 1:1000 for Gram-negative bacteria to get a concentration of $10^6$ colony forming units (CFU)/mL. Microtiter plates (96 wells of 100 µL) were filled peptide solution to obtain serial 2-fold dilution series of peptides. 50 µL of pure water was first added to each well. To the first well 50 µL peptide solution was added, after mixing 50 µL of this was transferred to the second well and so on. After all plates were prepared, 50 µL bacterial solution in double-concentration MHM was added to the wells (except for the final row of each plate) to give a final concentration of $5 \times 10^5$ CFU/mL in the wells. Thus, peptides got in contact with phosphate and bacteria simultaneously. This was found to be important, since the KL peptides aggregate stronlgy in contact with the phosphate containing growth medium [3]. The two final rows of each plate remained without peptide, so that the penultimate data point served as the positive control (no peptide), and the final one as the negative control (not inoculated). The plates were incubated at 37°C for 20 h, and cell viability was probed by addition of 20 µL 0.2 mg/mL resazurin and incubation at 37°C for 2 h. The MIC value was determined visually as the lowest peptide concentration inhibiting bacterial growth.

*1.4. Hemolysis assay*

Hemolytic activity was examined with a serial 2-fold dilution assay as described earlier [4]. Citrate phosphate dextrose-stabilized blood bags with erythrocyte suspensions of healthy donors were obtained from the blood bank of the local municipal hospital (Städtisches Klinikum, Karlsruhe, Germany). The erythrocytes, previously washed, were incubated with peptide solutions at 37°C for 30 min with gentle shacking. The tubes were centrifuged at 13000 rpm for 10 min to pellet the cells, and the absorbance at 540 nm was recorded against a negative control (cells without peptide, accounting for autohemolysis). The percentage of lysis was then calculated relative to 100% lysis induced by 1% Triton X-100. The absorbance measurements were repeated three times, and the averaged values were used.

*1.5. Vesicle leakage assays*

For the leakage experiments [5,6], the buffer in which the vesicles were prepared contained the fluorophore ANTS (12.5 mM), the quencher DPX (45 mM), 50 mM NaCl, and 10 mM HEPES (pH 7.5). Liposomes were prepared by co-dissolving PC/PG (1/1 mol/mol) mixtures of the lipids with different chain length in $CHCl_3$/MeOH (3/1 vol/vol), together with $10^{-2}$ mol% Rhod-PE by which the lipid loss during vesicle preparation (extrusion and gel filtration, see below) could be quantified. The peptide-to-lipid ratio (P/L) refers to a mol/mol ratio. As a reference for all samples, the P/L ratio was calculated for KL14, and the same peptide-to-lipid mass ratios were used for the other peptides in order to keep the total weight of peptidic material constant. The lipid mixture was dried under $N_2$(g) and left to dry under vacuum overnight. The obtained thin film was then re-suspended in buffer, which contained the fluorophore and the quencher, by vigorous vortexing, followed by 10 freeze-thaw cycles [7]. Large unilamellar vesicles (LUV) were obtained by 41-fold extrusion (Avanti Mini Extruder; Avanti Polar Lipids, Alabaster, AL) of the liposomes through a Nuclepore polycarbonate membrane (pore size 100 nm, Whatman - GE Healthcare Europe, Freiburg, Germany) at a temperature 20°C above the lipid phase transition. Unencapsulated dye was removed by gel filtration using spin columns filled with Sephacryl 100-HR (Sigma-Aldrich, Taufkirchen, Germany), and equilibrated with an elution buffer (150 mM NaCl, 10 mM HEPES, pH 7.5), which balances the internal vesicle osmolarity.

Leakage of entrapped ANTS was monitored by fluorescence dequenching of ANTS [8]. Fluorescence measurements were performed in a thermostated cuvette with constant stirring at 30°C in elution buffer, on a FluoroMax2 spectrofluorimeter (HORIBA Jobin Yvon, Unterhaching, Germany). The ANTS emission wavelength was 515 nm (5 nm slit) and the excitation wavelength was 355 nm (4 nm slit). The vesicles (100 µM lipid final concentration) of the desired composition were added to the cuvette containing the peptide at the P/L ratio to be tested. The level of 0% leakage corresponded to the fluorescence of the vesicles immediately after their addition, while 100% leakage was the fluorescence value obtained upon addition of Triton-X100 after 10 min.

### *1.6. Leakage of FITC-dextrans*

The size of holes in the membrane induced by the peptides was investigated using a modified leakage assay based on the fluorescence quenching of FITC-labeled dextran polymers with different size [9,10]. In short, POPC/POPG (1:1) vesicles were prepared with carboxyfluorescein and different size fluorescein isothiocyanate (FITC)-dextrans (FDs, Sigma-Aldrich, Taufkirchen, Germany) inside. When the FDs leak out, the fluorescence signal is quenched by anti-FITC antibodies (SouthernBiotech, USA). Leakage was followed for 30 minutes, then Triton-X100 was added to completely destroy the vesicles and get 100% leakage.

### *1.7. Size determination of FITC-dextrans*

The size of the FITC-dextrans was determined by dynamic light scattering (Zetasizer Nano S, Malvern Instruments Ltd.) as described previously [9,11]. The particle size was characterized at 25°C. According to the cumulants analysis the mean size (z-average diameter) of FITC-dextrans was derived from the slope of the linearized form of the autocorrelation function (first Cumulant). The results are shown in **Figure S1** and **Table S1**.

The sizes found are similar to the Stokes radii for FITC-dextrans provided by the supplier, which are approximately 1.4 nm for FD4, 3.3 nm for FD20, 4.5 nm for FD40, and 6.0 nm for FD70 [12].

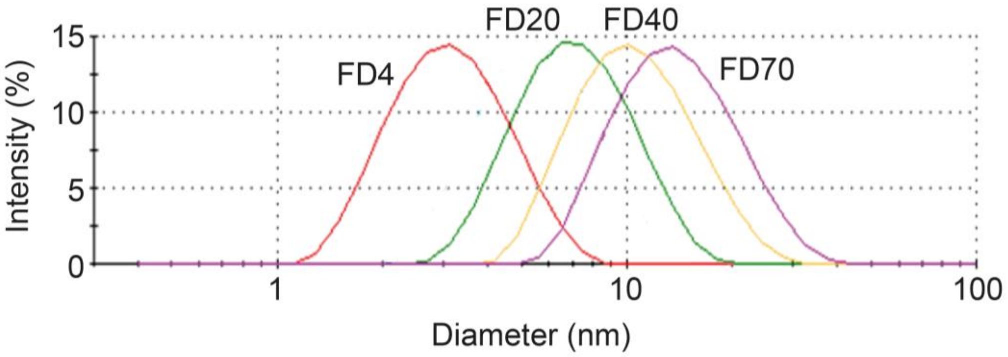


**Figure S1.** Results of size determination of FITC-dextrans by dynamic light scattering.

**Table S1.** Size of the FITC-dextrans according to dynamic light scattering measurements, assuming spherical particles. The size of carboxyfluorescein (CF) is taken from [13].

| Fluorophore | Molecular weight (Da) | Radius (nm) |
|---|---|---|
| CF | 376 | 0.63 |
| FD4 | 4000 | 1.65 |
| FD20 | 20000 | 3.7 |
| FD40 | 40000 | 5.6 |
| FD70 | 70000 | 7.45 |

*1.8. Solid-state NMR*

Macroscopically oriented NMR samples were prepared by co-dissolving appropriate amounts of peptides and lipids (in 300 µL methanol, 100 µL $CHCl_3$, and 10-20 µL milliQ-water), and spreading the solution onto 23 thin glass plates of dimensions 18 mm × 7.5 mm × 0.08 mm (Marienfeld Laboratory Glassware, Lauda-Königshofen, Germany). The peptide-to-lipid ratio (P/L) is given in mol/mol. For all samples and sample types, the P/L ratios were calculated for KL14 and the same peptide-to-lipid mass ratios were used for the other peptides to keep the charge ratio constant. The plates were dried in air for 1 h, followed by vacuum overnight. They were stacked and placed into a hydration chamber with 96% relative humidity at 48ºC for 18–24 h, before wrapping the stack in parafilm and plastic foil for the NMR measurements.

All solid-state NMR measurements were carried out on a Bruker Avance 500 or 600 MHz spectrometer (Bruker Biospin, Karlsruhe, Germany) at 308 K, as previously reported [14-19]. $^{31}$P-NMR was used to check the quality of the lipid orientation in the samples, using a Hahn echo sequence with phase cycling [20]. $^{1}$H-$^{15}$N cross polarization experiments using a CP-MOIST pulse sequence [21] were performed using a double-tuned probe with a low-E flat-coil resonator (3 mm × 9 mm cross section), employing a $^{1}$H and $^{15}$N radiofrequency field strength of 65 kHz during the cross polarization, and 36 kHz $^{1}$H SPINAL16 [22] decoupling during acquisition. A mixing time of 500 µs was used, and 10000 to 30000 scans were accumulated. The acquisition time was 10 ms and the recycle time 4 s. The $^{15}$N chemical shift was referenced using the signal of an ammonium sulfate sample set to 26.8 ppm. The oriented membrane samples were placed in the flat-coil probe such that the lipid bilayer normal was usually aligned parallel to the magnetic field, and additional experiments were carried out in a perpendicular alignment. $^{19}$F-NMR was performed using a home-built probe head at a frequency of 470.6 MHz, using an anti-ringing sequence [23] with a 90° pulse of 3.25 µs, a sweep width of 500 kHz, 4096 data points, and proton decoupling using TPPM [24]. Depending on the amount of peptide, between 10000 and 240000 scans were acquired. The $^{1}$H chemical shift was referenced using the $^{1}$H-NMR water signal of the sample, and the $^{19}$F chemical shift was calculated from the known gyromagnetic ratios of $^{1}$H and $^{19}$F.

## 2. Supplementary Table

**Table S2.** Hemolysis (in %) at different KL peptide concentrations (µg/mL). Values are compared with the values after addition of triton-X, which leads to 100% hemolysis. For KL14, KL16 and longer peptides the hemolysis is no longer increasing continuously with peptide concentration, probably due to aggregation.

| **Peptide** | **Concentration (µg/ml)** | | | | | | | | |
|---|---|---|---|---|---|---|---|---|---|
| | **1** | **2** | **4** | **8** | **16** | **32** | **64** | **128** | **256** |
| KL6 | 0 | 1 | 1 | 3 | 2 | 2 | 4 | 6 | 7 |
| KL8 | 2 | 5 | 7 | 9 | 9 | 10 | 12 | 16 | 21 |
| KL9 | 5 | 6 | 8 | 9 | 9 | 11 | 14 | 18 | 20 |
| LK9 | 7 | 13 | 14 | 16 | 19 | 25 | 33 | 47 | 66 |
| KL10 | 11 | 12 | 16 | 20 | 28 | 40 | 59 | 81 | 94 |
| LK10 | 6 | 14 | 15 | 18 | 22 | 25 | 29 | 34 | 50 |
| KL11 | 5 | 8 | 10 | 9 | 12 | 15 | 20 | 29 | 43 |
| LK11 | 17 | 39 | 63 | 87 | 99 | 97 | 98 | 98 | 100 |
| KL12 | 9 | 19 | 31 | 48 | 72 | 86 | 94 | 98 | 100 |
| KL13 | 9 | 22 | 33 | 46 | 64 | 81 | 90 | 93 | 100 |
| LK13 | 46 | 60 | 64 | 79 | 84 | 87 | 90 | 89 | 92 |
| KL14 | 47 | 86 | 96 | 96 | 95 | 94 | 94 | 91 | 89 |
| KL15 | 29 | 55 | 74 | 86 | 93 | 94 | 97 | 97 | 99 |
| LK15 | 57 | 73 | 73 | 78 | 81 | 84 | 82 | 81 | 79 |
| KL16 | 37 | 59 | 59 | 69 | 68 | 51 | 41 | 35 | 32 |
| KL18 | 33 | 80 | 95 | 95 | 90 | 82 | 79 | 78 | 78 |
| KL20 | 56 | 77 | 80 | 76 | 59 | 37 | 30 | 29 | 31 |
| KL22 | 58 | 87 | 87 | 77 | 53 | 49 | 49 | 50 | 48 |
| KL24 | 57 | 94 | 94 | 92 | 81 | 81 | 82 | 84 | 87 |
| KL26 | 52 | 71 | 72 | 72 | 56 | 50 | 47 | 42 | 39 |

## 3. Supplementary Figures

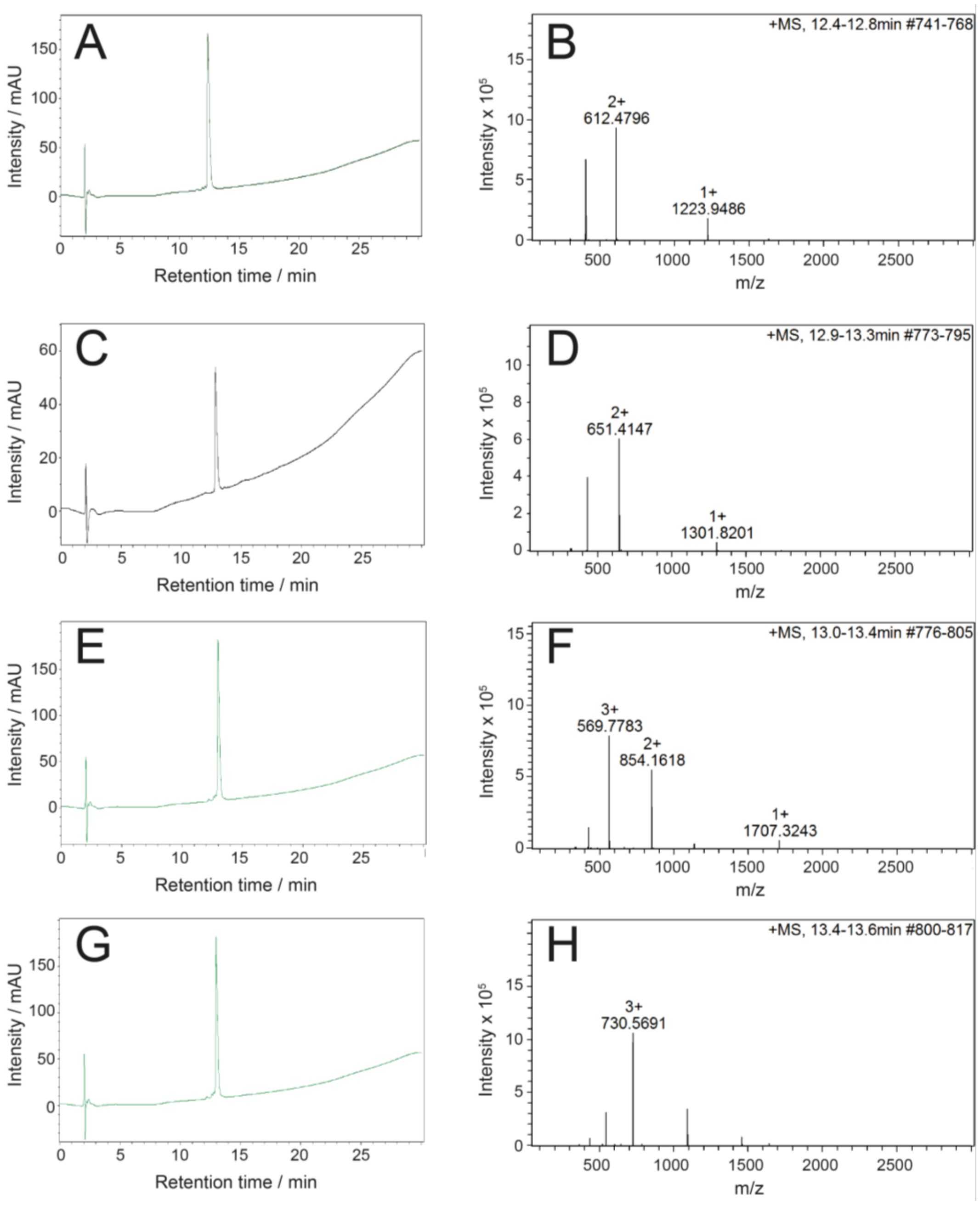


**Figure S2.** LC-MS chromatograms showing absorbance at 220 nm (A,C,E,G) and mass spectra (B,D,F,H) of selected peptides used in this study. In all cases there is a single peak in the chromato-gram and the expected mass is found for each peptide. (A,B) KL10, (C,D) KL10-19F, (E,F) KL14, (G,H) KL18.

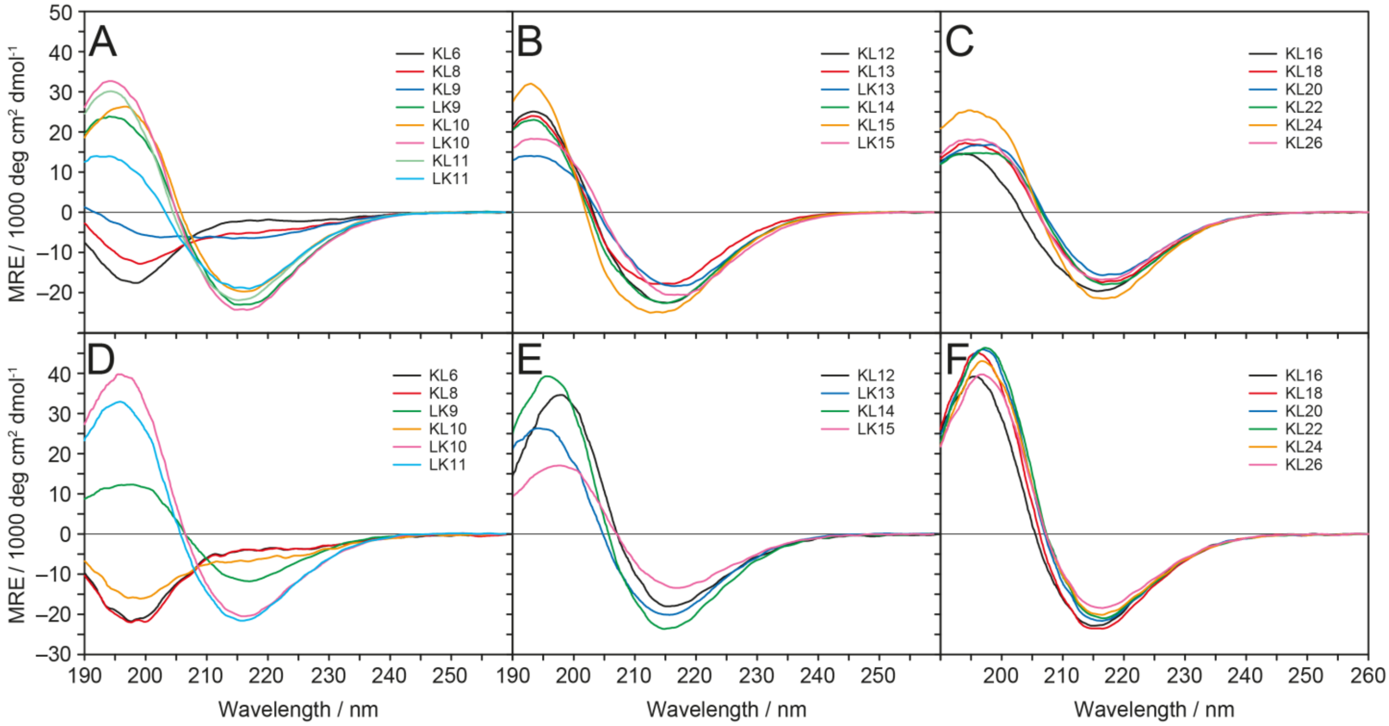


**Figure S3.** Circular dichroism spectra of KL and LK peptides. (A–C) CD spectra in pure water, at pH ≈ 9–10. The peptide concentration was 0.1 mg/mL. (D–F) CD spectra in the presence of DMPC SUVs at pH ≈ 9–10. The lipid concentration was 1 mg/mL (1.5 mM) and the peptide concentration for KL14 was 0.15 mg/mL (75 µM) resulting in a peptide-to-lipid (P/L) molar ratio of 1/20. The other samples were prepared in the same peptide-to-lipid mass-to-mass ratio to keep the charge ratio constant.

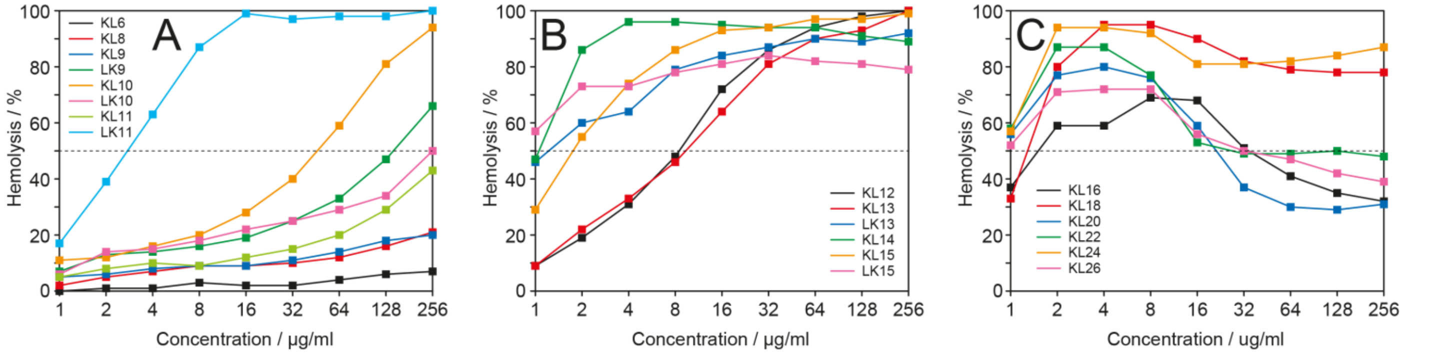


**Figure S4.** Hemolytic activity of KL and LK peptides. (A) Peptides with length 6–11 amino acids. (B) Peptides with length 12–15 amino acids. (C) Peptides with length 16–26 amino acids. In general, longer peptides are more active. For very long peptides, activity goes down at higher concentration, probably due to peptide aggregation. The dotted line in each panel indicates 50% hemolysis, and the HC50 value is determined from the crossing of the hemolysis curves with this line.

## Supplementary References


1. Wadhwani, P.; Strandberg, E.; Heidenreich, N.; Bürck, J.; Fanghänel, S.; Ulrich, A.S. Self-assembly of flexible β-strands into immobile amyloid-like β-sheets in membranes as revealed by solid-state $^{19}$F NMR. *J. Am. Chem. Soc.* **2012**, *134*, 6512-6515, doi:10.1021/Ja301328f.
2. Bürck, J.; Wadhwani, P.; Fanghänel, S.; Ulrich, A.S. Oriented circular dichroism: a method to characterize membrane-active peptides in oriented lipid bilayers. *Acc. Chem. Res.* **2016**, *49*, 184–192, doi:10.1021/acs.accounts.5b00346.
3. Strandberg, E.; Schweigardt, F.; Wadhwani, P.; Bürck, J.; Reichert, J.; Cravo, H.L.P.; Burger, L.; Ulrich, A.S. Phosphate-dependent aggregation of $[KL]_n$ peptides affects their membranolytic activity. *Sci. Rep.* **2020**, *10*, 12300, doi:10.1038/s41598-020-69162-0.

4. Strandberg, E.; Tiltak, D.; Ieronimo, M.; Kanithasen, N.; Wadhwani, P.; Ulrich, A.S. Influence of C-terminal amidation on the antimicrobial and hemolytic activities of cationic α-helical peptides. *Pure Appl. Chem.* **2007**, *79*, 717-728, doi:10.1351/pac200779040717.
5. Duzgunes, N.; Wilschut, J. Fusion assays monitoring intermixing of aqueous contents. *Methods Enzymol.* **1993**, *220*, 3-14, doi:10.1016/0076-6879(93)20069-F.
6. Steinbrecher, T.; Prock, S.; Reichert, J.; Wadhwani, P.; Zimpfer, B.; Bürck, J.; Berditsch, M.; Elstner, M.; Ulrich, A.S. Peptide-lipid interactions of the stress-response peptide TisB that induces bacterial persistence. *Biophys. J.* **2012**, *103*, 1460-1469, doi:10.1016/j.bpj.2012.07.060.
7. Mayer, L.D.; Hope, M.J.; Cullis, P.R. Vesicles of variable sizes produced by a rapid extrusion procedure. *Biochim. Biophys. Acta* **1986**, *858*, 161-168, doi:10.1016/0005-2736(86)90302-0.
8. Ellens, H.; Bentz, J.; Szoka, F.C. $H^+$- and $Ca^{2+}$-induced fusion and destabilization of liposomes. *Biochemistry* **1985**, *24*, 3099-3106, doi:10.1021/Bi00334a005.
9. Stutzin, A. A fluorescence assay for monitoring and analyzing fusion of biological membrane-vesicles in vitro. *FEBS Lett.* **1986**, *197*, 274-280, doi:10.1016/0014-5793(86)80341-6.
10. Ladokhin, A.S.; Selsted, M.E.; White, S.H. Sizing membrane pores in lipid vesicles by leakage of co-encapsulated markers: pore formation by melittin. *Biophys. J.* **1997**, *72*, 1762-1766, doi:10.1016/S0006-3495(97)78822-2.
11. Reichert, J.; Grasnick, D.; Afonin, S.; Bürck, J.; Wadhwani, P.; Ulrich, A.S. A critical evaluation of the conformational requirements of fusogenic peptides in membranes. *Eur. Biophys. J.* **2007**, *36*, 405-413, doi:10.1007/s00249-006-0106-2.
12. Sigma-Aldrich. Fluorescein Isothiocyanate-Dextran. Available online: https://www.sigmaaldrich.com/technical-documents/protocols/biology/fluorescein-isothiocyanate-dextran.html#ref (accessed on 14 July 2022).
13. Wooten, M.K.C.; Koganti, V.R.; Zhou, S.S.; Rankin, S.E.; Knutson, B.L. Synthesis and nanofiltration membrane performance of oriented mesoporous silica thin films on macroporous supports. *ACS Appl. Mater. Inter.* **2016**, *8*, 21806-21815, doi:10.1021/acsami.6b06765.
14. Grage, S.L.; Strandberg, E.; Wadhwani, P.; Esteban-Martin, S.; Salgado, J.; Ulrich, A.S. Comparative analysis of the orientation of transmembrane peptides using solid-state $^2$H- and $^{15}$N-NMR: mobility matters. *Eur. Biophys. J.* **2012**, *41*, 475-482, doi:10.1007/s00249-012-0801-0.
15. Müller, S.D.; De Angelis, A.A.; Walther, T.H.; Grage, S.L.; Lange, C.; Opella, S.J.; Ulrich, A.S. Structural characterization of the pore forming protein $TatA_d$ of the twin-arginine translocase in membranes by solid-state $^{15}$N-NMR. *Biochim. Biophys. Acta* **2007**, *1768*, 3071-3079, doi:10.1016/j.bbamem.2007.09.008.
16. Glaser, R.W.; Sachse, C.; Dürr, U.H.N.; Afonin, S.; Wadhwani, P.; Strandberg, E.; Ulrich, A.S. Concentration-dependent realignment of the antimicrobial peptide PGLa in lipid membranes observed by solid-state $^{19}$F-NMR. *Biophys. J.* **2005**, *88*, 3392-3397, doi:10.1529/biophysj.104.056424.
17. Strandberg, E.; Zerweck, J.; Wadhwani, P.; Ulrich, A.S. Synergistic insertion of antimicrobial magainin-family peptides in membranes depends on the lipid spontaneous curvature. *Biophys. J.* **2013**, *104*, L9-11, doi:10.1016/j.bpj.2013.01.047.
18. Heinzmann, R.; Grage, S.L.; Schalck, C.; Bürck, J.; Banoczi, Z.; Toke, O.; Ulrich, A.S. A kinked antimicrobial peptide from *Bombina maxima*. II. Behavior in phospholipid bilayers. *Eur. Biophys. J.* **2011**, *40*, 463-470, doi:10.1007/s00249-010-0668-x.
19. Walther, T.H.; Grage, S.L.; Roth, N.; Ulrich, A.S. Membrane alignment of the pore-forming component $TatA_d$ of the twin-arginine translocase from *Bacillus subtilis* resolved by solid-state NMR spectroscopy. *J. Am. Chem. Soc.* **2010**, *132*, 15945-15956, doi:10.1021/ja106963s.
20. Rance, M.; Byrd, R.A. Obtaining high-fidelity spin-1/2 powder spectra in anisotropic media - phase-cycled Hahn echo spectroscopy. *J. Magn. Reson.* **1983**, *52*, 221-240, doi:10.1016/0022-2364(83)90190-7.
21. Levitt, M.H.; Suter, D.; Ernst, R.R. Spin dynamics and thermodynamics in solid-state NMR cross polarization. *J. Chem. Phys.* **1986**, *84*, 4243-4255, doi:10.1063/1.450046.
22. Fung, B.M.; Khitrin, A.K.; Ermolaev, K. An improved broadband decoupling sequence for liquid crystals and solids. *J. Magn. Reson.* **2000**, *142*, 97-101, doi:10.1006/jmre.1999.1896.
23. Zhang, S.; Wu, X.L.; Mehring, M. Elimination of ringing effects in multiple-pulse sequences. *Chem. Phys. Lett.* **1990**, *173*, 481-484, doi:10.1016/0009-2614(90)87239-N.
24. Bennett, A.E.; Rienstra, C.M.; Auger, M.; Lakshmi, K.V.; Griffin, R.G. Heteronuclear decoupling in rotating solids. *J. Chem. Phys.* **1995**, *103*, 6951-6958, doi:10.1063/1.470372.
